# A comparative analysis of complete chloroplast genomes of seven *Ocotea* species (Lauraceae) confirms low sequence divergence within the *Ocotea* complex

**DOI:** 10.1038/s41598-021-04635-4

**Published:** 2022-01-21

**Authors:** Dimitrij Trofimov, Daniel Cadar, Jonas Schmidt-Chanasit, Pedro Luís Rodrigues de Moraes, Jens G. Rohwer

**Affiliations:** 1 Institute of Plant Science and Microbiology, Universität Hamburg, Ohnhorststraße 18, 22609 Hamburg, Germany; 2 Institute of Ecology and Evolution, Universität Jena, Philosophenweg 16, 07743 Jena, Germany; 3 Bernhard Nocht Institute for Tropical Medicine, Bernhard-Nocht-Straße 74, 20359 Hamburg, Germany; 4 Departamento de Botânica, Instituto de Biociências, Universidade Estadual Paulista “Júlio de Mesquita Filho”, Av. 24 A 1515, Bela Vista, Rio Claro, Caixa Postal 199, São Paulo, CEP 13506-900 Brazil

**Keywords:** Genome, Plant genetics, Plant molecular biology

## Abstract

The genus *Ocotea* (Lauraceae) includes about 450 species, of which about 90% are Neotropical, while the rest is from Macaronesia, Africa and Madagascar. In this study we present the first complete chloroplast genome sequences of seven *Ocotea* species, six Neotropical and one from Macaronesia. Genome sizes range from 152,630 (*O. porosa*) to 152,685 bp (*O. aciphylla*). All seven plastomes contain a total of 131 (114 unique) genes, among which 87 (80 unique) encode proteins. The order of genes (if present) is the same in all Lauraceae examined so far. Two hypervariable loci were found in the LSC region (*psbA-trnH*, *ycf2*), three in the SSC region (*ycf1*, *ndhH*, *trnL(UAG)-ndhF*). The pairwise cp genomic alignment between the taxa showed that the LSC and SSC regions are more variable compared to the IR regions. The protein coding regions comprise 25,503–25,520 codons in the *Ocotea* plastomes examined. The most frequent amino acids encoded in the plastomes were leucine, isoleucine, and serine. SSRs were found to be more frequent in the two dioecious Neotropical *Ocotea* species than in the four bisexual species and the gynodioecious species examined (87 vs. 75–84 SSRs). A preliminary phylogenetic analysis based on 69 complete plastomes of Lauraceae species shows the seven *Ocotea* species as sister group to *Cinnamomum* sensu lato. Sequence divergence among the *Ocotea* species appears to be much lower than among species of the most closely related, likewise species-rich genera *Cinnamomum*, *Lindera* and *Litsea*.

## Introduction

The Lauraceae are among the most frequent woody plant families in moist tropical areas and include about 55 genera with 2500–3500 species^1–3^. The genus *Ocotea* Aubl., in its current circumscription, is the largest genus among the Neotropical Lauraceae, consisting of about 400–450 recognized species^2–8^. The number of Paleotropical *Ocotea* species is far smaller. The majority (34 spp.) is endemic to Madagascar, four are found in Continental Africa, three on Mauritius, one on Réunion Island, and one on the Comoro islands. *Ocotea foetens* (Aiton) Baill. is endemic to Macaronesia^3,7^.

Most of the molecular phylogenetic studies in Lauraceae published so far focused on other genera or on the major evolutionary lineages in the Lauraceae and included only a relatively small number of *Ocotea* species^9–11^. Nevertheless, they suggested that *Ocotea* was paraphyletic with respect to most other New World genera, viz. *Aniba* Aubl., *Damburneya* Raf., *Dicypellium* Nees & Mart., *Endlicheria* Nees, *Kubitzkia* van der Werff, *Licaria* Aubl., *Nectandra* Rol. ex Rottb., *Paraia* Rohwer, H.G. Richt. & van der Werff, *Pleurothyrium* Nees, *Rhodostemonodaphne* Rohwer & Kubitzki, *Umbellularia* (Nees) Nuttall and *Urbanodendron* Mez. A recent study based on RAD-seq data^12^ added *Phyllostemonodaphne* Kosterm. to this list. These genera, plus presumably *Gamanthera* van der Werff and *Povedadaphne* W.C. Burger, which have not been studied yet, are collectively referred to as the *Ocotea* complex^10^ or Supraocotea^12^, a group of about 950 species. A higher number of *Ocotea* species than in previous studies, plus representative species of other genera of the *Ocotea* complex, were studied by Trofimov et al.^2^ and Trofimov and Rohwer^3^, with similar results. Using sequences of the nuclear internal transcribed spacer (ITS) and one of the most informative parts of the chloroplast genome, the *psbA-trnH* spacer, they separated two genera from *Ocotea* s. lat., namely *Mespilodaphne* Nees & Mart. and *Kuloa* Trofimov & Rohwer. In addition, several of the morphological groups described by Rohwer^4^ were confirmed as monophyletic in these studies. Resolution and/or support values at the lower nodes within the *Ocotea* complex, however, remained poor. Most of the other established chloroplast markers tested in the research group of the senior author [JGR] (*atpB-rbcL*, *matK*, *ndhF-rpl32*, *psbK-psbI, rbcL, rpl16*, *rpb2*, *rpl3–trnL*, *rpl32-trnL*, *rpoB*, *rpoC1*, *trnG–trnS*, *trnL-trnF*, and *trnT-trnL*) turned out to be less informative in molecular analyses of the *Ocotea* complex, or problematic because of too many single nucleotide repeats. Therefore, no significant improvement was to be expected from sequencing individual chloroplast markers any more. Sequencing of entire chloroplast (cp) genomes, on the other hand, is expected to yield a higher number of informative characters, which will probably lead to better support for the lower nodes within the *Ocotea* complex. The present study of selected *Ocotea* plastomes is intended as a first step towards this goal. The most recent phylogeny by Penagos Zuluaga et al.^12^ based on RAD-seq data is fully resolved at the lower nodes, with strong bootstrap support for all of the basal and most of the more distal nodes, so that it will provide an ideal basis for comparison with our and future cp genome data.

The chloroplast (cp) genome is a circular molecule ranging in size from 107 to 218 kb. It shows a characteristic quadripartite structure with a pair of inverted repeats (IRs) separating a large single copy (LSC) and a small single copy (SSC) region^13,14^. The typical angiosperm cp genome consists of 120–130 genes, coding mainly for RNAs and photosynthesis-related genes^15^.

Up to the present, the plastomes of Lauraceae were studied mainly in Asian species of *Actinodaphne* Nees, *Alseodaphne* Nees, *Beilschmiedia* Nees, *Cryptocarya* R. Br., *Caryodaphnopsis* Airy Shaw, *Cassytha* L., *Cinnamomum* Schaeff., *Dehaasia* Blume, *Endiandra* R. Br., *Eusideroxylon* Teijsm. & Binn., *Iteadaphne* Blume, *Laurus* L., *Lindera* Thunb., *Litsea* Lam., *Machilus* Nees, *Neocinnamomum* H. Liu, *Neolitsea* (Benth. & Hook. f.) Merr., *Nothaphoebe* Blume, *Parasassafras* D.G. Long, *Phoebe* Nees*, Sassafras* J. Presl and *Syndiclis* Hook. F.^16–33^. Neotropical species were poorly represented in previous studies of the cp genome. Only *Nectandra angustifolia* (Schrad.) Nees & Mart. (but see below) and *Persea americana* Mill. have been studied so far, plus the North American *P. borbonia* (L.) Spreng.^24,25,28^. These studies considerably improved support values among the major phylogenetic lineages in the Asian Lauraceae, especially among *Cassytha*, *Caryodaphnopsis* and *Neocinnamomum*. In other plant groups, such as the genus *Quercus* L., Poaceae-Arundinarieae and Rosaceae, they allowed resolving phylogenetic relationships on different levels^34–36^.

In this study, we sequenced and analyzed the complete chloroplast genomes of six Neotropical and the only Macaronesian *Ocotea* species using Illumina high-throughput sequencing technology. This is the first study of this kind in this species-rich and ecologically important group. We describe the structure of the plastomes examined, amino acid percentage of protein-coding genes, content of Simple Sequence Repeats (SSRs), relative synonymous codon usage for protein coding nucleotides and variability values in the *Ocotea* plastomes, and compare them to 85 plastomes of Lauraceae. In addition, we performed a preliminary phylogenetic analysis to show the positions of the seven *Ocotea* species among 62 plastomes of Core Lauraceae in the sense of Rohwer and Rudolph^37^ examined so far, i.e. Cinnamomeae, Laureae and Perseeae. The *Ocotea* complex forms the largest clade within the Cinnamomeae, with the Laureae and the Perseeae as consecutive sister groups. However, this paper does not have a phylogenetic focus but rather provides basic data on chloroplast genomes that may be used in future phylogenetic studies.

## Results

### Organization of the plastomes of *Ocotea*

**Figure 1:**
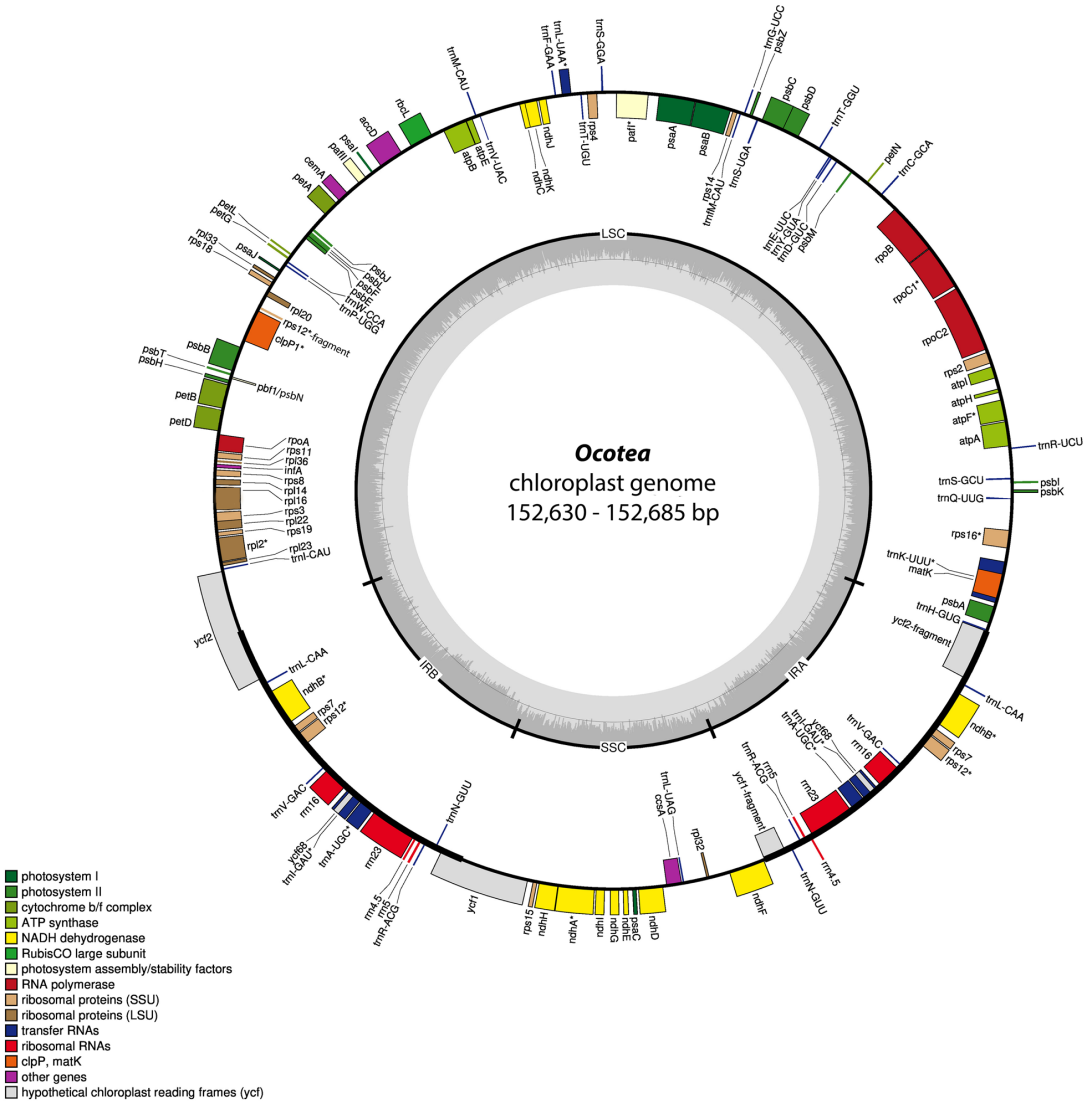
Gene map of *Ocotea s*pecies (*O. aciphylla*, *O. daphnifolia*, *O. foetens*, *O. guianensis*, *O. odorifera*, *O. porosa*, and *O. tabacifolia*) chloroplast genomes. The genes shown on the inside and the outside of the outer circle are transcribed in clockwise and counterclockwise direction, respectively. The coloured bars denote gene functional groups. The dark gray and light gray shading within the inner circle correspond to percentage GC and AT content, respectively. *IR* inverted repeat, *LSC* large single copy, *SSC* small single copy.

The chloroplast genome sequences of the seven *Ocotea* species range from 152,630 bp in *O. porosa* (Nees & Mart.) Barroso to 152,685 bp in *O. aciphylla* (Nees & Mart.) Mez (Table 1). The plastomes show the typical quadripartite structure of chloroplast genomes. Two inverted repeat (IR) regions (20,009–20,015 bp) are separated by a large single copy (LSC) region (93,815–93,859 bp) and a small single copy (SSC) region (18,775–18,818 bp) (Fig. 1, Table 1). All seven *Ocotea* plastomes contain a total of 131 genes (114 unique), among which 87 (80 unique) encode proteins (Table 2). The order of genes (if present) is the same in all Lauraceae so far examined. Fourteen genes have one intron (*atpF*, *ndhA*, *ndhB*, *rpl2*, *rpl16*, *rpoC1*, *rps12*, *rps16*, *trnA-UGC*, *trnG-UCC*, *trnI-GAU*, *trnK-UUU*, *trnL-UAA*, and *trnV-UAC*), and two (*clpP* and *pafI*) have two introns (Table 2, Supplementary Table S1). The total GC content in plastomes is same in all *Ocotea* species examined (39.2%; Table 1). Contents of nucleotides in the LSC, IR and SSC of the plastomes were similar in all species of *Ocotea* examined (Supplementary Table S2). About 30.3–30.4%, 27.3–28.3%, and 32.9% were detected for A; 19.3–19.4%, 21.1–23.4%, and 21.1% for C; 18.6%, 21.0–23.4%, and 18.1% for G; and 31.6–31.7%, 27.2–28.3%, and 33.1% for T, respectively. The GC content in the IR regions was higher than in the LSC and SSC regions (44.4%, vs. 37.9–38.0% and 33.9–34.0%, respectively).

**Table 1:** Summary of seven complete plastomes of *Ocotea* species.

	*O. aciphylla*	*O. daphnifolia*	*O. foetens*	*O. guianensis*	*O. odorifera*	*O. porosa*	*O. tabacifolia*
Raw reads no	2,290,010	2,161,228	5,898,677	8,358,607	4,373,153	1,985,942	2,103,610
Clean reads no.	2,287,092	2,143,358	5,896,116	8,355,240	4,370,826	1,984,720	2,102,190
Gene no. (unique)	131 (114)	131 (114)	131 (114)	131 (114)	131 (114)	131 (114)	131 (114)
Protein coding genes no. (unique)	87 (80)	87 (80)	87 (80)	87 (80)	87 (80)	87 (80)	87 (80)
tRNA genes no. (unique)	36 (30)	36 (30)	36 (30)	36 (30)	36 (30)	36 (30)	36 (30)
rRNA genes no. (unique)	8 (4)	8 (4)	8 (4)	8 (4)	8 (4)	8 (4)	8 (4)
cp genome length, bp	152,685	152,635	152,656	152,656	152,646	152,630	152,652
Large single copy (LSC) length, bp	93,849	93,834	93,859	93,858	93,841	93,815	93,851
Inverted repeat (IR) length, bp	20,009	20,009	20,011	20,009	20,009	20,015	20,009
Small single copy (SSC) length, bp	18,818	18,783	18,775	18,780	18,787	18,785	18,783
Protein coding (CDS) length, bp	76,577	76,591	76,591	76,567	76,591	76,559	76,576
Total GC content (%)	39.2	39.2	39.2	39.2	39.2	39.2	39.2

**Table 2:** Genes encoded by seven *Ocotea* plastomes.

Category	Group of genes	Name of genes
Photosynthesis related genes	Rubisco	*rbcL*
	Photosystem I	*psaA*, *psaB*, *psaC*, *psaI*, *psaJ*
	Assembly/stability of photosystem I	pafI**, *pafII*
	Photosystem II	*psbA*, *psbB*, *psbC*, *psbD*, *psbE*, *psbF*, *psbH*, *psbI*, *psbJ*, *psbK*, *psbL*, *psbM*, *psbN, psbT, inhA/psbZ*
	ATP synthase	*atpA*, *atpB*, *atpE*, atpF*, *atpH*, *atpI*
	Cytochrome b/f complex	*petA*, *petB*, *petD*, *petG*, *petL*, *petN*
	Cytochrome c synthesis	*ccsA*
	NADH-dehydrogenase	ndhA*, ndhB* (2x), *ndhC*, *ndhD*, *ndhE*, *ndhF*, *ndhG*, *ndhH*, *ndhI*, *ndhJ*, *ndhK*
Transcription and translation related genes	Subunit of RNA polymerase	*rpoA*, *rpoB*, rpoC1*, *rpoC2*
	Ribosomal protein small subunit	*rps2*, *rps3*, *rps4*, *rps7* (2x), *rps8*, *rps11*, rps12* (2x), *rps12-fragment*, *rps14*, *rps15*, rps16*, *rps18*, *rps19*
	Ribosomal protein large subunit	rpl2*, *rpl14*, rpl16*, *rpl20*, *rpl22, rpl23*, *rpl32*, *rpl33*, *rpl36*
	Translation initiation factor	*infA*
RNA genes	Ribosomal RNAs	*rrn4*.*5* (2x), *rrn5* (2x), *rrn16* (2x), *rrn23* (2x)
	Transfer RNAs	*trnA*-UGC* (2x), *trnC-GCA*, *trnD*-*GUC*, *trnE*-*UUC*, *trnF*-*GAA*, *trnG*-*GCC, trnG*-UCC*, *trnH*-*GUG*, *trnI*-*CAU*, *trnI*-GAU* (2x), *trnK*-UUU*, *trnL-CAA* (2x), *trnL*-UAA*, *trnL*-*UAG*, *trnfM-CAU*, *trnM*-*CAU*, *trnN*-*GUU* (2x), *trnP*-*UGG*, *trnQ*-*UUG*, *trnR*-*ACG* (2x), *trnR*-*UCU*, *trnS*-*GCU*, *trnS*-*GGA*, *trnS*-*UGA*, *trnT*-*GGU*, *trnT*-*UGU*, *trnV*-*GAC* (2x), trnV-UAC*, *trnW*-*CCA*, *trnY*-*GUA*
Miscellaneous group	Maturase	*matK*
	Envelope membrane protein	*cemA*
	Subunit of Acetyl-CoA-Carboxylase	*accD*
	Proteolysis	clpP**
Genes of unknown function	Conserved open reading frames	*ycf1*, *ycf1-fragment, ycf2*, *ycf2 -fragment*
Putative pseudogenes		*ycf68* (2x)

(2x)—Gene represented by two copies.

*Gene containing one intron.

**Gene containing two introns.

### Determination of the most variable regions

**Figure 2:**
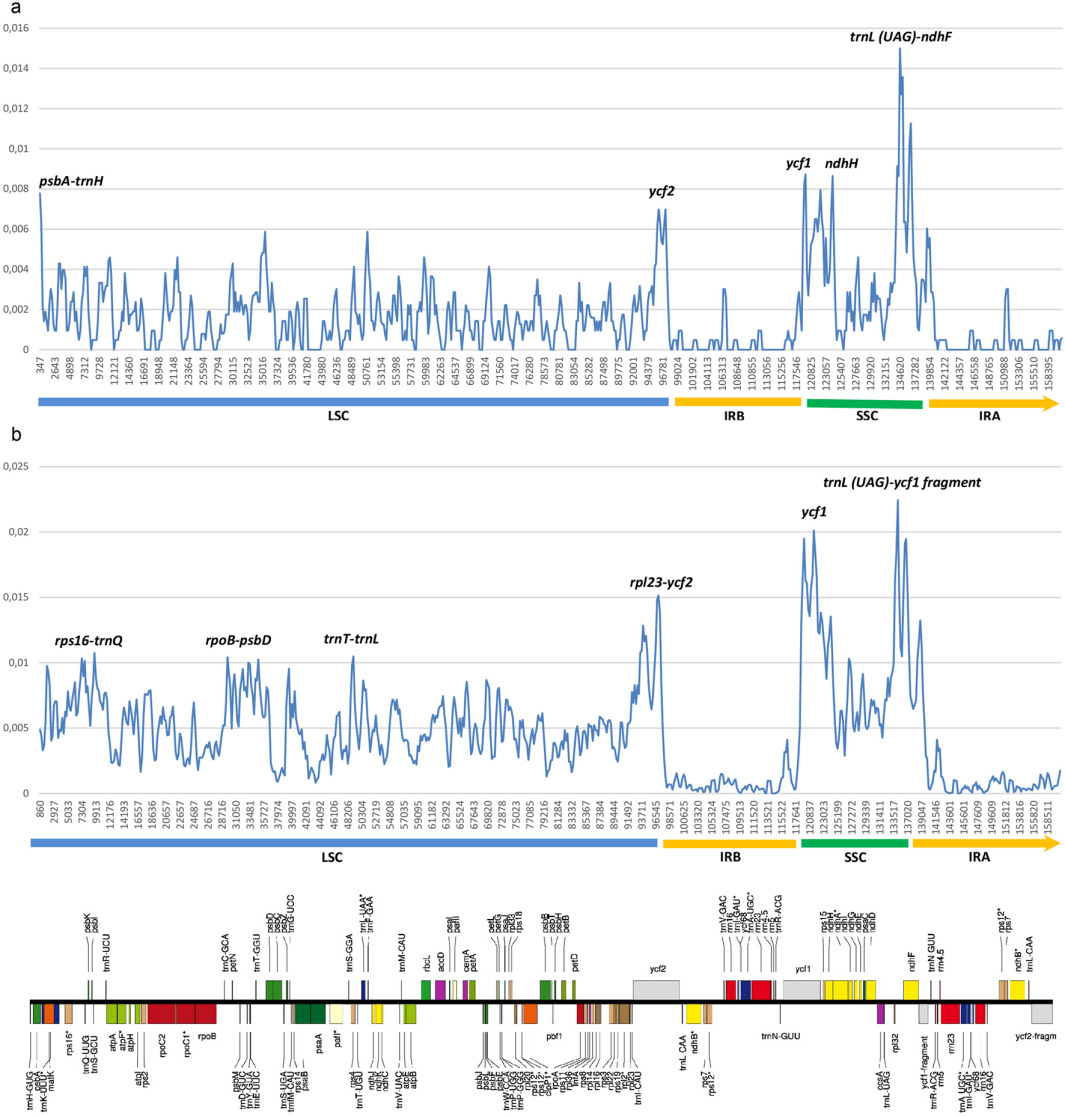
Comparison of the nucleotide variability (Pi) values, (**a**) among the seven *Ocotea* plastomes and (**b**) among 69 plastomes of Lauraceae. The linear gene map of *Ocotea* species is shown below.

The nucleotide diversity (Pi) values within 600 bp across the seven *Ocotea* plastomes vary from 0 to 0.015, with a mean value of 0.001 (Fig. 2a). Four variable loci with Pi ≥ 0.006 were found in the LSC region (*psbA-trnH*, Pi = 0.007; *ycf2*, Pi = 0.006) and in the SSC region (*ycf1*, Pi = 0.008; *ndhH*, Pi = 0.008; *trnL(UAG)-ndhF*, Pi = 0.015). At the family level, sequence divergence was calculated using published chloroplast genomes of *Alseodaphne, Cinnamomum, Laurus, Lindera, Litsea, Machilus, Neolitsea, Parasassafras, Persea, Phoebe,* and *Sassafras* (see “Materials and methods” section). Unfortunately, the sequence of *Nectandra angustifolia* (marked as “unverified” in GenBank) had to be excluded because it differs so strongly from those of all other Core Lauraceae that large parts of it could not be readily aligned. The Pi values among the 69 plastomes vary from 0 to 0.022, with a mean value of 0.0045 (Fig. 2b). Variable loci with Pi > 0.01 were identified in the LSC region (*rps16-trnQ*, Pi = 0.01; *rpoB-psbD*, Pi = 0.01; *trnT-trnL*, Pi = 0.01; *rpl23-ycf2*, Pi = 0.014) and in the SSC region (*ycf1*, Pi = 0.019; *trnL(UAG)-ycf1*, Pi = 0.022). The open reading frames *ycf1* and *ycf2* are located in one of the IR regions (IRb), at the border of the SSC and the LSC region, respectively.

### Comparative analysis of plastomes

**Figure 3:**
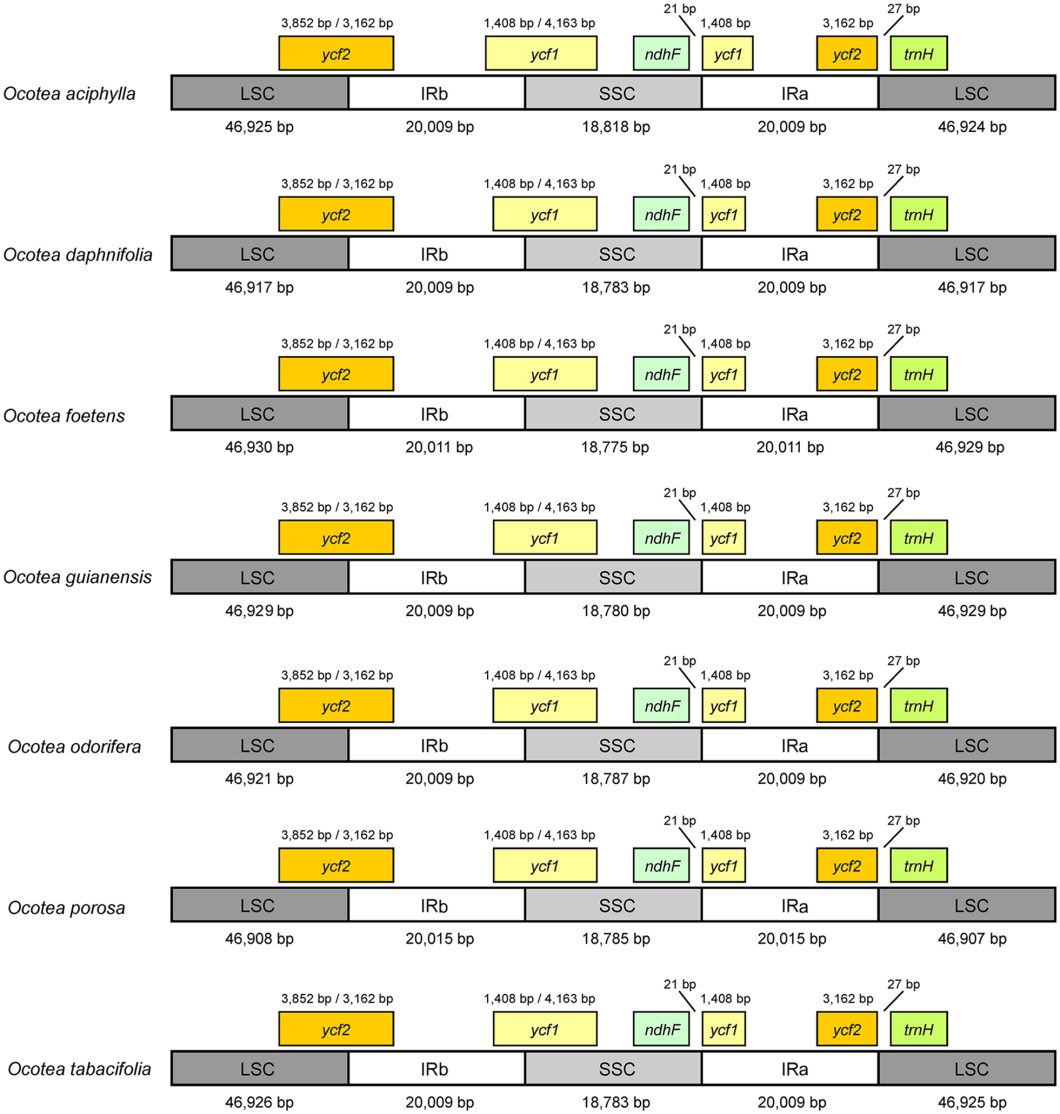
Comparison of LSC, IR, and SSC junction positions among seven *Ocotea* chloroplast genomes.

**Figure 4:**
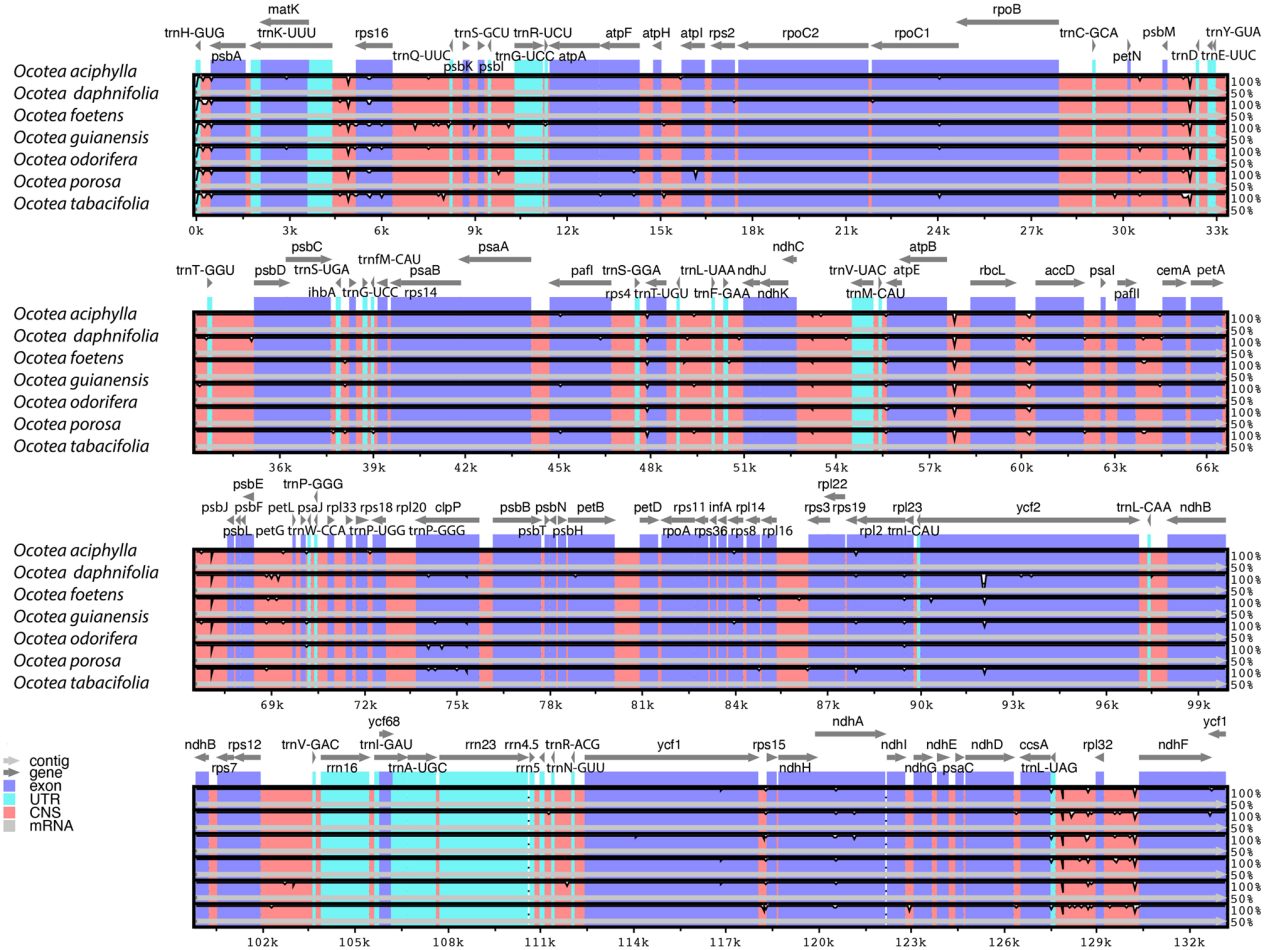
Visualization of *Ocotea* chloroplast genomes using mVISTA program with *O. aciphylla* as reference. *CNS* conserved non-coding sequence; *UTR* untranslated region.

A comparison of the LSC, IR and SSC junction positions in the *Ocotea* plastomes is shown in Fig. 3. The *ycf1* gene crosses the boundary between the IRb (1408 bp) and the SSC (4163 bp) regions. The *ycf2* gene is found in the boundary between the LSC (3852 bp) and the IRb (3162 bp) regions. Fragments (pseudogenes) of *ycf1* (1408 bp) and *ycf2* (3162 bp) are located in the IRa region. The distances between the *ndhF* gene and the *ycf1* fragment and between the *ycf2*-fragment and the *trnH* gene are 21 bp and 27 bp, respectively. The pairwise cp genomic alignment between six *Ocotea* species and *O. aciphylla* as reference showed very high similarity in all sequences (Fig. 4). The LSC and SSC regions were more variable in comparison with the IR regions. The noncoding regions showed a relatively higher mutation rate than protein-coding regions in the *Ocotea* plastomes examined.

### Codon usage analysis

**Figure 5:**
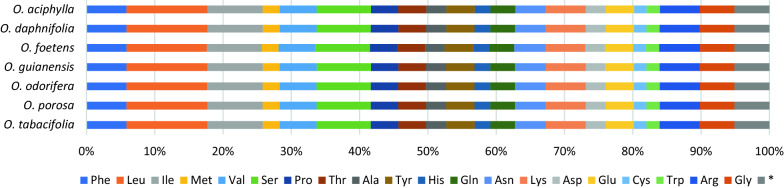
Amino acid percentage of protein-coding genes in seven *Ocotea* plastomes.

The count of codons in the plastoms examined here were 25,503–25,520 with an average number of about 25,514 (Supplementary Tables S3, S4). The effective number of codons (ENC), Codon Bias Index (CBI) as well the Scaled Chi-square (SChi2) were very similar in all *Ocotea* plastomes (56.59–56.62; 0.15; 0.073–0.074, respectively) (Supplementary Table S4). The GC content at coding positions is about 39.1% in the examined *Ocotea* plastomes. The GC contents at second and at third codon positions were also very similar (35.5–35.6%; 39.2% respectively). All possible codon types are used for each amino acid. The most frequent amino acids encoded in the *Ocotea* plastomes are leucine (Leu; 11.76–11.83%), isoleucine (Ile; 8.05%–8.11%), and serine (Ser; 7.93–8.01%) (Fig. 5). The amino acids arginine (Arg), glycine (Gly), lysine (Lys), phenylalanine (Phe), and valine (Val) account for 5.02–5.95% each. Least represented in the chloroplast genomes examined were cysteine (Cys; 1.81–1.87%) and tryptophan (Trp; 1.94–1.95%). The relative synonymous codon usage (RSCU) was greater than 1.0 in 31 codons (Supplementary Table S3). The count of preferred codons ending with A/U or G/C were 25 and six, respectively. The frequency of different codons coding for the same amino acid was almost the same in all *Ocotea* species examined. The Macaronesian *Ocotea foetens* presented slightly higher frequencies for arginine, cysteine, serine, histidine (His), and tyrosine (Tyr) in comparison with the Neotropical *Ocotea* species, whereas the contents of alanine (Ala), isoleucine and leucine were slightly lower.

### Simple sequence repeats (SSRs) analysis

**Figure 6:**
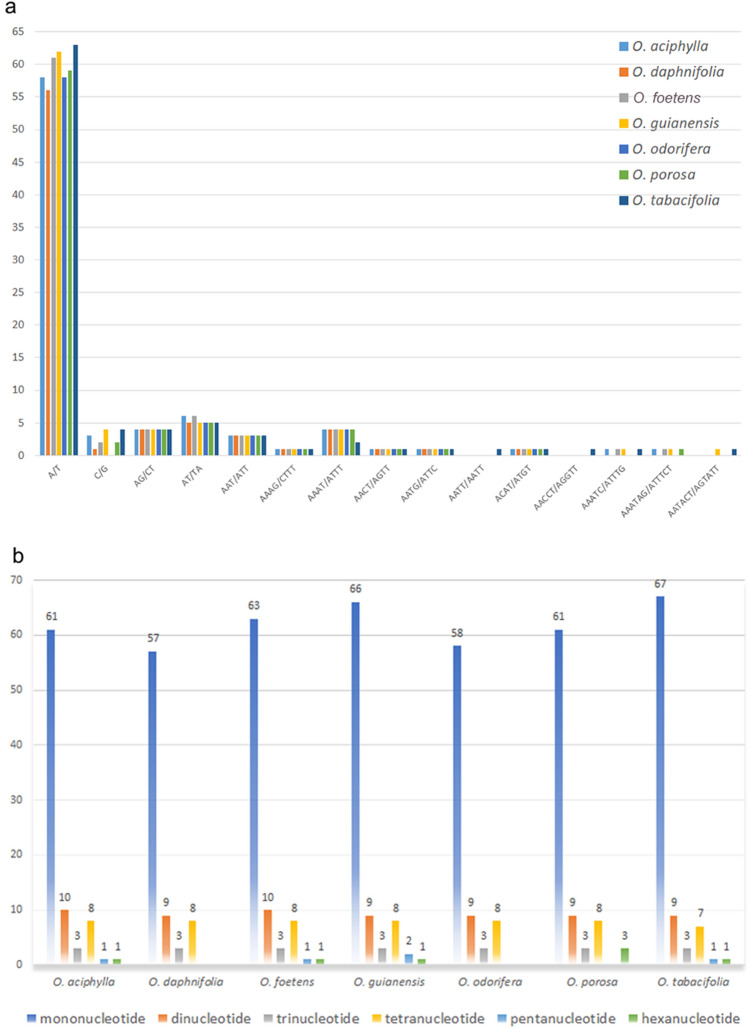
Simple sequence repeats (SSRs) in seven chloroplast genomes of *Ocotea*. (**a**) Counts of nucleotide repeats; (**b**) counts of mono-, di-, tri-, tetra-, penta- and hexanucleotides.

The seven chloroplast genomes examined showed a total 586 SSRs with a repeat length of one to six bp (Fig. 6a, Supplementary Table S5). These SSRs were mainly mononucleotide repeats (433 SSRs = 74%) of A or T (417), less frequently C or G (16). In addition, there were 65 dinucleotide repeats (11%), 21 tri- (4%), 55 tetra- (9%), five penta- (1%), and seven hexanucleotide repeats (1%). The numbers of SSRs observed in the different *Ocotea* species were relatively similar. In each plastome we identified 77–89 SSRs, incl. 57–67 mono-, nine or ten di-, three tri-, seven or eight tetra-, zero, one or two penta-, and zero, one or three hexanucleotide repeats (Fig. 6b). The SSRs were identified mainly in the LSC region (62–70 SSRs; Supplementary Table S5), compared to one or two and 11–17 SSRs in the IR and SSC regions, respectively.

### Phylogenetic analysis of Lauraceae plastomes

**Figure 7:**
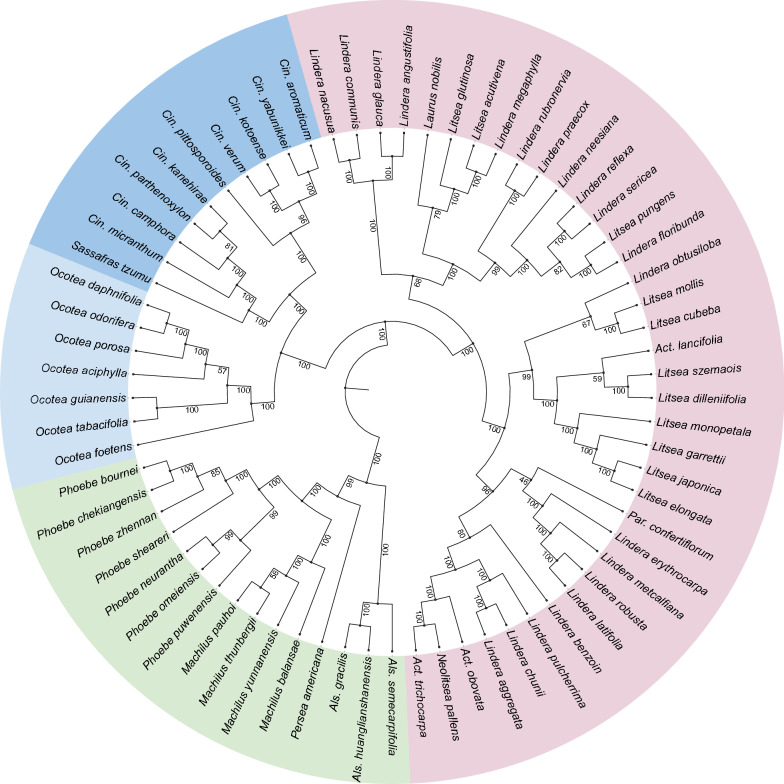
Maximum Likelihood phylogeny including 69 complete chloroplast genome sequences of Core Lauraceae. Color codes: blue—Cinnamomeae, incl. pale blue for *Ocotea* spp.; pink—Laureae; green—Perseeae. Maximum likelihood bootstrap support values (ML-BS) are shown next to the branches.

The data matrix consisted of 160,629 characters, among which 5266 were variable but parsimony-uninformative, and 4631 were parsimony-informative. However, only 168 characters were parsimony-informative among the seven *Ocotea* species in this analysis. Most clades in the Maximum Likelihood analysis received 100% bootstrap support (ML-BS, Fig. 7, Supplementary Fig. S1). With the Perseeae defined as the outgroup, Laureae and Cinnamomeae are shown as sister clades in the ingroup. Among the Cinnamomeae, species of *Cinnamomum*, with *Sassafras* nested among them, form the sister group to the seven *Ocotea* species examined here. The Macaronesian *Ocotea foetens* is shown as sister taxon to the six Neotropical species. Among these, the two dioecious species, *Ocotea guianensis* Aubl. and *O. tabacifolia* (Meisn.) Rohwer, form the sister group to the remaining species, which are bisexual or gynodioecious [*O. daphnifolia* (Meisn.) Mez]. The latter clade, however, is barely supported (57% ML-BS). *Ocotea aciphylla* (Nees & Mart.) Mez appears as sister taxon to the remaining species, and among these *O. porosa* (Nees & Mart.) Barroso is shown as sister taxon to *O. daphnifolia* and *O. odorifera* (Vell.) Rohwer.

## Discussion

The genome sizes of the *Ocotea* species examined in this study are similar to those of other Core Lauraceae^16–33^, as well as *Caryodaphnopsis* and *Neocinnamomum* species^25^ (Supplementary Table S6). The genomes of Cryptocaryeae (*Beilschmiedia, Cryptocarya, Endiandra, Eusideroxylon* and *Syndiclis*) are more than 5000 bp larger^25,28^. The cp genome of the hemiparasitic *Cassytha*, on the contrary, is ca. 40,000 bp smaller than those of the Core Lauraceae^25^. *Cassytha* has lost not only its functional *ndh* genes, like many hemiparasitic plants^38^, but also an entire inverted repeat region.

The seven *Ocotea* chloroplast genomes show some length variation in all of their parts (LSC, IRs, SSC). Consistently smaller length variation was found among the species of *Alseodaphne* (3 spp.), *Endiandra* (4 spp.), *Neocinnamomum* (2 spp.), *Phoebe* (3 spp.) and *Syndiclis* (2 spp.) so far examined^25–28^. Larger variation was found among the species of *Beilschmiedia* (6 spp.), *Caryodaphnopsis* (3 spp.), *Cinnamomum* (7 spp.), *Litsea* (14 spp.) and *Persea* (3 spp.)^16,17,24,25,28–30^. However, the intrageneric differences are expected to increase, also in *Ocotea*, with increased number of species examined. It is therefore too early to make statements about the relative length variability in different clades. Surprisingly large intrageneric differences of about 6000 bp in total chloroplast, LSC and IR regions, and about 400 bp in the SSC region were observed among *Caryodaphnopsis* species, *C. henryi, C. malipoensis* and *C. tonkinensis*^25,28^.

A total of 131 (114 unique) genes were identified in the *Ocotea* species examined. For most Lauraceae examined so far (species of *Actinodaphne*, *Alseodaphne*, *Beilschmiedia, Caryodaphnopsis*, *Cinnamomum*, *Cryptocarya*, *Eusideroxylon*, *Lindera*, *Machilus*, *Nectandra*, *Neocinnamomum*, *Neolitsea*, *Persea*, *Phoebe* and *Sassafras*), the number of genes was indicated as 128–130 (113 unique)^23–27,33^. Lower numbers (127 total/112 unique) were reported for some species of *Actinodaphne, Cinnamomum*, *Lindera, Litsea,* and *Neolitsea*^17,29,30^, but only 107 genes (total and unique) in two *Cassytha* species^25^.

A total of 87 protein coding genes were identified in the *Ocotea* species examined. The counts of total protein coding genes in Lauraceae in previous studies ranged from 73 genes in *Cassytha* species via 79 in *Cinnamomum camphora* to 86 genes in *Caryodaphnopsis henryi* Airy Shaw^16,25,30^. Consistently 85 protein coding genes have been reported for the genera of the early divergent Cryptocaryeae (*Beilschmiedia, Cryptocarya* and *Eusideroxylon*), as far as they have been examined. Among the remaining Lauraceae, the most frequent count is 84^30^. Lower numbers have been reported for *Cinnamomum micranthum* and *C. kanehirae* (83)^29^, *Litsea glutinosa* (83)^17^, *Cinnamomum camphora* (79)^16^ and two *Cassytha* species (73)^25^. The differences among the counts of genes in the Lauraceae species, except the hemiparasitic *Cassytha*, may be due to different annotation of genes. Particularly the *rpl22* gene has not been annotated in most of the earlier studies^17,19,23,24,26,27,29,30,33,39–41^.

The *psbA-trnH*, *ycf1*, *ycf2*, *ndhH* and *trnL(UAG)-ndhF* regions were identified as hypervariable loci (Pi ≥ 0.006) at the species level among the *Ocotea* species examined here. Seven hypervariable regions (Pi > 0.014), *ihbA-trnG, ndhA, ndhF-rpl32, psbK-psbI, rps16, trnS-trnG* and *ycf1* were identified in *Lindera* species^33^. The *psbA-trnH*, *ycf2* and *ndhH* regions are not among the most variable regions in these species. *Alseodaphne* species show six hypervariable loci (Pi > 0.006), *accD-psaI, ndhF-rpl32, rps19-rpl3, rpl32-trnL, trnG-UCC,* and *ycf1*^26^*.* Seven hypervariable loci (Pi > 0.008), *clpP*, *ndhF-rpl32, rpl32-trpL, rps8-rpl14, trnQ-psbI, ycf1*, and *ycf2*, were identified in *Machilus* species^23^. At the family level, we identified additional hypervariable regions (Pi ≥ 0.01) among 69 Core Lauraceae species, viz. *rpoB-psbD* and *trnT-trnL*. Zhao et al.^33^ detected only *ndhF-rpl32* and *ycf1* as hypervariable loci (Pi > 0.014) among the Core Lauraceae. By comparing the *Ocotea* plastomes using the mVISTA program^42,43^, we confirmed that the IR regions are more conservative than the LSC and SSC regions. The LSC and SSC regions comprise more noncoding regions with higher mutation rates. The protein-coding sequences, including 80 genes, were longer in the *Ocotea* plastomes than in the plastome of *Cinnamomum camphora* (76,509–76,560 bp = 25,503–25,520 codons vs. 63,654 bp = 21,218 codons)^16^.

In our study and in Chen et al.^16^ the codons coding for leucine and for cysteine were the most and the least frequent, respectively. 11.76–11.83% of the codons in *Ocotea* and 10.87% in *Cinnamomum camphora* are coding for leucine, whereas only 1.81–1.87% or 1.25%, respectively, are coding for cysteine. Like in *C. camphora*, preferred codons in *Ocotea* are more frequently ending in A/U than in G/C (27 vs. two in *C. camphora*, 25 vs. six in *Ocotea*).

Simple sequence repeats (SSRs) are widely distributed in chloroplast genomes of Lauraceae. Chen et al.^16^ detected 81, 82, 83–88, and 86 SSRs in *Litsea, Machilus, Cinnamomum* and *Persea* species, respectively. In this study, we found more SSRs in the two Neotropical dioecious *Ocotea* species examined, *O. guianensis* and *O. tabacifolia*, than in the other four Neotropical species (87 vs. 75–82 SSRs), which are bisexual or gynodioecious (*O. daphnifolia*). The plastome of the Macaronesian *Ocotea foetens* contains 84 SSRs. It remains to be checked if the number of SSRs is indeed correlated with larger clades within the *Ocotea* complex. Mononucleotide SSRs are very predominant in the chloroplast sequences of Lauraceae. The counts of mononucleotide SSRs varied from 54 to 65 in *Litsea, Machilus, Cinnamomum* and *Persea* species^16^. In *Ocotea*, we detected 57–67 mononucleotide SSRs. Among the Neotropical *Ocotea* species, we found the highest numbers in the two dioecious species (66–67, vs. 57–61 in the other four species). The Macaronesian *Ocotea foetens* showed 63 mononucleotide SSRs. Hexanucleotide repeats were rare in all Lauraceae species examined so far. No hexanucleotide SSRs were found in *Ocotea daphnifolia* and *O. odorifera*. However, we detected three hexanucleotide repeats in *Ocotea porosa*, instead of only one in most other Lauraceae. The numbers of SSRs in the LSC, SSC and IR regions were similar for all Lauraceae species studied. Chen et al.^16^ detected 63, 16 and four SSRs in the LSC, SSC and IR regions of *Cinnamomum camphora* vs. 62–70, 11–17 and one or two SSRs in the *Ocotea* species in our study.

As expected, addition of the seven *Ocotea* species does not change the result of the phylogenetic analysis significantly compared to previous cp genome studies^28,33^. The topology among the major clades, Cinnamomeae, Laureae and the Perseeae, is the same in all studies. As excpected, the seven *Ocotea* species form a monophyletic group that is sister to *Cinnamomum* s.lat., i.e., including *Sassafras*. It is unfortunate that the cp genome of the taxon recorded as ‘UNVERIFIED *Nectandra angustifolia*’ in GenBank (MF939340) is so divergent from all other Core Lauraceae that large parts of it could not even be aligned. Based on the results of earlier studies^2,3,9,10,12^, *Nectandra* was expected to be nested in *Ocotea*, as sister taxon to the dioecious clade. Not only because of its aberrant sequence it is questionable if the species listed as *N. angustifolia* in the study of Song et al.^25^ has been determined correctly. The real *N. angustifolia* is known from the type collection from Bahia only, so that it appears unlikely that it was cultivated in Sulawesi. Apart from *Nectandra angustifolia*, no complete plastomes have been sequenced so far in any of the genera that are usually found nested among the *Ocotea* species (*Aniba*, *Damburneya*, *Dicypellium*, *Endlicheria*, *Kubitzkia*, *Licaria*, *Mespilodaphne, Nectandra*, *Paraia*, *Pleurothyrium*, *Rhodostemonodaphne*, *Umbellularia* and *Urbanodendron*). The number of *Ocotea* species examined here is still too small to reach any definite conclusions about their phylogeny. There are, however, two differences compared to the recent study by Penagos et al.^12^. In their study, the Old World *Ocotea* species (the clade named Palaeocotea) form the sister group to a clade named Praelicaria, which is represented by *Ocotea aciphylla*, *O. odorifera* and *O. porosa* in our study. *Ocotea daphnifolia*, which is nested among the Praelicaria taxa in our result, is a member of the *O. minarum* group and as such a member of the Pluriocotea clade in the study by Penagos et al.^12^. In their result, the Pluriocotea clade is the sister group to a clade consisting of the dioecious taxa (Diocotea, represented by *O. guianensis* and *O. tabacifolia* in our study), the *O. helicterifolia* group and the genera *Nectandra*, *Pleurothyrium* and *Damburneya*, which are not represented in our study. It needs to be checked if these differences persist when further cp genomes become available. As expected, entire plastomes have the potential to increase resolution and support values among the clades of the *Ocotea* complex. Our phylogeny is fully resolved, and not only the *Ocotea* complex receives 100% bootstrap support, but also four of the five nodes within it. There is still one node that is scarcely supported, but that may change with denser taxon sampling.

Sequence divergence among the seven *Ocotea* species is rather low, compared to the most closely related, likewise species-rich genera *Cinnamomum*, *Lindera* and *Litsea*. Even though we selected *Ocotea* species from widely divergent clades, there were only 168 parsimony-informative characters among them in the entire chloroplast genomes. If we arbitrarily select the first seven species of *Cinnamomum*, *Lindera* or *Litsea* from our data matrix, these numbers are 414, 423 or 410, respectively. This confirms the results of the tests of individual established chloroplast markers mentioned in the introduction, and may point to a rather recent diversification of the *Ocotea* complex, as was first suggested by Chanderbali et al.^10^. However, a much larger number of sequences will be required for a molecular clock analysis of this group.

## Materials and methods

### Plant materials

Silica-gel dried leaf material of seven *Ocotea* species, *O. aciphylla*, *O. daphnifolia*, *O. foetens*, *O. guianensis*, *O. odorifera*, *O. porosa*, and *O. tabacifolia*, was used for the present analysis (Supplementary Table S7). According to previous analyses^2,3,12^, these species belong to different clades within the genus, except *Ocotea odorifera* and *O. porosa* from the *O. indecora* group. The plant material was collected in accordance with the relevant institutional, national, and international guidelines and legislation. PLRM obtained the collecting permits for the material collected in Brazil 2011. *Ocotea foetens* was collected in the Botanical Garden of Berlin, with permission of the curator G. Parolly, from a tree of unknown origin that had been growing in the garden for decades. Voucher specimens are deposited in the herbarium Rioclarense (HRCB) at the Universidade Estadual Paulista, Rio Claro (Brazil), the herbarium Hamburgense (HBG) at the University of Hamburg (Germany), and the garden herbarium of the Botanical Garden and Botanical Museum Berlin (Germany).

### DNA preparation and chloroplast sequencing

DNA was isolated with the innuPREP Plant DNA Kit (Analytik Jena, Germany) according to the manufacturer’s protocol, with modifications^9,37^. DNA libraries were built using the QIAseq FX DNA Library Kit (Qiagen, Germany) and 120 ng of each DNA. Normalized samples were pooled and sequenced using the 300-cycles (2 × 150 bp paired-end) MiSeq reagent kit v3 (Illumina, San Diego, CA) on a MiSeq platform at the NGS Core Facility at the Bernhard Nocht Institute for Tropical Medicine, Hamburg, Germany. The generated raw reads were first checked qualitatively, with Phred quality score < 20 trimmed and filtered to remove polyclonal and low quality reads (< 55 bases long) using CLC workbench v. 20.0.1 (Qiagen).

### Plastomes assembly and annotation

Analyses of genome sequence and genomic organization were performed using Geneious Prime 2021.0.3^44^. The generated contigs of *Ocotea foetens* were assembled de novo and annotated using the plastomes of *Cinnamomum camphora* (GenBank accession number MH050970) and *Persea americana* (NC_031189) for comparison. The contigs of the remaining taxa were assembled and annotated using the chloroplast genome of *O. foetens* as a reference. The contigs were inspected visually for any signs of erroneous assembly. In a few cases, doubtful regions were verified by Sanger sequencing (methods described earlier^2,3,9,11^). The circular plastome maps of *Ocotea* were drawn using OGDRAW v1.2^29,33–38,44–47^.

### Determination of the most variable regions of plastomes

The chloroplast genomes of seven *Ocotea* species and 63 other Lauraceae were downloaded from the NCBI GenBank (Supplementary Table S8) All 70 sequences were aligned using MAFFT v7^48^ with default parameters. Visual inspection of the alignment showed that large parts of the sequence of *Nectandra angustifolia* could not be aligned with confidence, so that this species had to be removed. Ten small inversions (5–39 base pairs), bordered by long palindromic sequences, were identified and reversed, because earlier analyses had shown that the orientation of such hairpin loops varies even within a single population. In the final alignment, these inversions correspond to positions 272–276, 480–487, 29,786–29,810, 66,770–66,809, 69,509–69,522, 70,298–70,314, 117,696–117,701 (in *Parasassafras* only), 126,376–126,385, 132,449–132,505 and 140,474–140,479 (in *Parasassafras* only). Also a few additional minor adjustments of the alignment were made manually during inspection of the sequences, mostly in regions of SSRs. DnaSP v6^49^ was used for calculating the nucleotide variability values (Pi) within the plastomes. The sliding window length was set to 600 bp, and the step size was set to 200 bp. Microsoft Excel^50^ was used to plot the Pi values. These data were used to identify hypervariable regions among the seven *Ocotea* plastomes examined as well as among the sequences retrieved from the NCBI GenBank (Supplementary Tables S7, S8).

### Comparative analysis of *Ocotea* plastomes

A comparison of the LSC, IR and SSC junction positions in the *Ocotea* plastomes was carried out in Geneious Prime 2021.0.3^44^. The mVISTA program in Shuffle-LAGAN mode^42,43^ was used for the visualization of the differences in the seven *Ocotea* chloroplast genomes.

#### Codon usage and SSRs analyses

The protein-coding genes of *Ocotea* plastoms were extracted using the program Geneious Prime 2021.0.3^44^. The sequences were aligned using MAFFT v7^48^. Codon usage frequency, Codon Bias, and G + C content were calculated using the program DnaSP v6.

The SSR motifs were scanned using MISA v2.1^51^. The minimum thresholds were set to 10 repetitions for mononucleotide SSRs, five repeat units for dinucleotide SSRs, four repetitions for trinucleotide SSRs and three repetitions for tetra-, penta- and hexanucleotide SSRs. The maximum length of interruption between two SSRs was chosen as 100 bp.

#### Phylogenetic analysis of Lauraceae plastomes

The data matrix that had been prepared for the determination of the most variable regions was analyzed using maximum likelihood analyses (ML) in MEGA 10.2.5^52^, with the following parameters: nrep = 500, Tamura-Nei model, uniform rates among sites and Nearest-Neighbor-Interchange (NNI). The chloroplast genomes of the Perseeae (*Alseodaphne* spp., *Machilus* spp., *Persea americana*, and *Phoebe* spp.) were used as outgroup.

## Supplementary Information

Supplementary Information.

## Data Availability

The complete cp genome sequences of the seven *Ocotea* species have been submitted to the NCBI GenBank.

## References

[1] Rohwer JG, Kubitzki K (1993). Lauraceae. The Families and Genera of Vascular Plants.

[2] Trofimov D, Moraes PLR, Rohwer JG (2019). Towards a phylogenetic classification of the *Ocotea* complex (Lauraceae)—Classification principles and reinstatement of *Mespilodaphne*. Bot. J. Linn. Soc..

[3] Trofimov D, Rohwer JG (2020). Towards a phylogenetic classification of the *Ocotea* complex (Lauraceae)—An analysis with emphasis on the Old World taxa and description of the new genus *Kuloa*. Bot. J. Linn. Soc..

[4] Rohwer JG (1986). Prodromus einer Monographie der Gattung *Ocotea* Aubl. (Lauraceae), sensu lato. Mitt. Inst. Allg. Bot. Hamburg.

[5] van der Werff H (1996). Studies in Malagasy Lauraceae II: New taxa. Novon.

[6] van der Werff H (2002). A synopsis of *Ocotea* (Lauraceae) in Central America and Southern Mexico. Ann. Missouri Bot. Gard..

[7] van der Werff H (2013). A revision of the genus *Ocotea* Aubl. (Lauraceae) in Madagascar and the Comoro Islands. Adansonia.

[8] van der Werff H (2017). Studies in Andean Ocotea (Lauraceae) IV Species with unisexual flowers and densely pubescent leaves, or with erect pubescence or domatia, occurring above 1000 m in altitude. Novon.

[9] Trofimov D, Rudolph B, Rohwer JG (2016). Phylogenetic study of the genus *Nectandra* (Lauraceae), and reinstatement of *Damburneya*. Taxon.

[10] Chanderbali AS, van der Werff H, Renner SS (2001). Phylogeny and historical biogeography of Lauraceae: Evidence from the chloroplast and nuclear genomes. Ann. Missouri Bot. Gard..

[11] Rohde R (2017). Neither *Phoebe* nor *Cinnamomum*—The tetrasporangiate species of *Aiouea* (Lauraceae). Taxon.

[12] Penagos Zuluaga JC (2021). Resolved phylogenetic relationships in the *Ocotea* complex (Supraocotea) facilitate phylogenetic classification and studies of character evolution. Am. J. Bot..

[13] Palmer JD (1985). Comparative organization of chloroplast genomes. Annu. Rev. Genet..

[14] Chumley TW (2006). The complete chloroplast genome sequence of *Pelargonium* x *hortorum*: Organization and evolution of the largest and most highly rearranged chloroplast genome of land plants. Mol. Biol. Evol..

[15] Ruhlman TA, Jansen RK (2014). The plastid genomes of flowering plants. Methods Mol. Biol..

[16] Chen C (2017). The complete chloroplast genome of *Cinnamomum camphora* and its comparison with related Lauraceae species. PeerJ.

[17] Hinsinger DD, Strijk JS (2017). Toward phylogenomics of Lauraceae: The complete chloroplast genome sequence of *Litsea glutinosa* (Lauraceae), an invasive tree species on Indian and Pacific Ocean islands. Plant Gene.

[18] Jo S, Kim YK, Cheon SH, Fan Q, Kim KJ (2019). Characterization of 20 complete plastomes from the tribe Laureae (Lauraceae) and distribution of small inversions. PLoS ONE.

[19] Liao Q, Ye T, Song Y (2018). Complete chloroplast genome sequence of a subtropical tree, *Parasassafras confertiflorum* (Lauranceae). Mitochondrial DNA B.

[20] Wang Q (2020). The complete chloroplast genome sequence of *Litsea cubeba*. Mitochondrial DNA B.

[21] Liao Q, Ye T, Song Y (2018). Complete chloroplast genome sequence of a subtropical tree, *Parasassafras confertiflorum* (Lauranceae [sic!]). Mitochondrial DNA B.

[22] Qiu Q, Yang D, Xu L, Xu Y, Wang Y (2020). The complete chloroplast genome sequence of *Litsea garrettii*. Mitochondrial DNA B Resour..

[23] Song Y (2015). Comparative analysis of complete chloroplast genome sequences of two tropical trees *Machilus yunnanensis* and *Machilus balansae* in the family Lauraceae. Front. Plant Sci..

[24] Song Y, Yao X, Tan Y, Gan Y, Corlett RT (2016). Complete chloroplast genome sequence of the avocado: Gene organization, comparative analysis, and phylogenetic relationships with other Lauraceae. Can. J. For. Res..

[25] Song Y (2017). Evolutionary comparisons of the chloroplast genome in Lauraceae and insights into loss events in the Magnoliids. Gen. Biol. Evol..

[26] Song Y, Yao X, Liu B, Tan Y, Corlett RT (2018). Complete plastid genome sequences of three tropical *Alseodaphne* trees in the family Lauraceae. Holzforschung.

[27] Song Y (2017). Comparative analysis of complete chloroplast genome sequences of two subtropical trees, *Phoebe sheareri* and *Phoebe omeiensis* (Lauraceae). Tree Genet. Genomes.

[28] Song Y (2020). Plastid phylogenomics improve phylogenetic resolution in the Lauraceae. J. Syst. Evol..

[29] Wu CC, Chu FH, Ho CK, Sung CH, Chang SH (2017). Comparative analysis of the complete chloroplast genomic sequence and chemical components of *Cinnamomum micranthum* and *Cinnamomum kanehirae*. Holzforschung.

[30] Xiao TW (2020). Conflicting phylogenetic signals in plastomes of the tribe Laureae (Lauraceae). PeerJ.

[31] Yuan X, Li Y, Wang Y (2019). The complete chloroplast genome sequence of *Cinnamomum kotoense*. Mitochondrial DNA B Resour..

[32] Zhang J, Li Y, Wang Y (2019). The complete chloroplast genome sequence of *Phoebe puwenensis*. Mitochondrial DNA B Resour..

[33] Zhao M-L (2018). Comparative chloroplast genomics and phylogenetics of nine *Lindera* species (Lauraceae). Sci. Rep..

[34] Ma PF, Zhang YX, Zeng CX, Guo ZH, Li DZ (2014). Chloroplast phylogenomic analyses resolve deep-level relationships of an intractable bamboo Tribe Arundinarieae (Poaceae). Syst. Biol..

[35] Yang YC (2016). Comparative analysis of the complete chloroplast genomes of five *Quercus* species. Front. Plant Sci..

[36] Zhang SD (2017). Diversification of Rosaceae since the late cretaceous based on plastid phylogenomics. New Phytol..

[37] Rohwer JG, Rudolph B (2005). Jumping genera: the phylogenetic positions of *Cassytha, Hypodaphnis,* and *Neocinnamomum* (Lauraceae) based on different analyses of trnK intron sequences. Ann. Missouri Bot. Gard..

[38] Shin HW, Lee NS (2018). Correction: Understanding plastome evolution in Hemiparasitic Santalales: Complete chloroplast genomes of three species, *Dendrotrophe varians, Helixanthera parasitica,* and *Macrosolen cochinchinensis*. PLoS ONE.

[39] Rabah SO (2017). Plastome Sequencing of ten nonmodel crop species uncovers a large insertion of mitochondrial DNA in cashew. Plant Genome..

[40] Li Y, Xu W, Zou W, Jiang D, Liu X (2017). Complete chloroplast genome sequences of two endangered *Phoebe* (Lauraceae) species. Bot. Stud..

[41] Liu D, Liu D, Li M, Chen S (2019). The complete chloroplast genome of *Phoebe zhennan*. Mitochondrial DNA B.

[42] Brudno M (2003). Comparative sequencing program. LAGAN and multi-LAGAN: Efficient tools for large-scale multiple alignment of genomic DNA. Genome Res..

[43] Frazer KA, Pachter L, Poliakov A, Rubin EM, Dubchak I (2004). VISTA: Computational tools for comparative genomics. Nucl. Acids Res..

[44] Kearse M (2012). Geneious basic: An integrated and extendable desktop software platform for the organization and analysis of sequence data. Bioinformatics.

[45] Tillich M (2017). GeSeq—Versatile and accurate annotation of organelle genomes. Nucl. Acids Res..

[46] Kent WJ (2002). BLAT—The BLAST-like alignment tool. Genome Res..

[47] Lohse M, Drechsel O, Bock R (2007). OrganellarGenomeDRAW (OGDRAW)—A tool for the easy generation of high-quality custom graphical maps of plastid and mitochondrial genomes. Curr. Genet..

[48] Katoh K, Rozewicki J, Yamada KD (2019). MAFFT online service: Multiple sequence alignment, interactive sequence choice and visualization. Brief. Bioinform..

[49] Rozas J (2017). DnaSP 6: DNA sequence polymorphism analysis of large data sets. Mol. Biol. Evol..

[50] Microsoft Excel. Microsoft Corporation (2018). Microsoft Excel.

[51] Beier S, Thiel T, Münch T, Scholz U, Mascher M (2017). MISA-web: A web server for microsatellite prediction. Bioinformatics.

[52] Kumar S, Stecher G, Li M, Knyaz C, Tamura K (2018). MEGA X: Molecular evolutionary genetics analysis across computing platforms. Mol. Biol. Evol..

