# Supplementary material for: A comparative analysis of complete chloroplast genomes of seven Ocotea species (Lauraceae) confirms low sequence divergence within the Ocotea complex
Source: Sci Rep. 2022 Jan 21;12:1120. doi: 10.1038/s41598-021-04635-4 (PMC8782842; doi:10.1038/s41598-021-04635-4)
Supplement: Supplementary file 1 — Supplementary Information. [file 41598_2021_4635_MOESM1_ESM.pdf]

Supplementary Information

A comparative analysis of complete chloroplast genomes of seven *Ocotea* species (Lauraceae) confirms low sequence divergence within the *Ocotea* complex

Dimitrij Trofimov[1,2], Daniel Cadar[3], Jonas Schmidt-Chanasit[3], Pedro Luís Rodrigues de Moraes[4] & Jens G. Rohwer[1]

[1]Universität Hamburg, Institute of Plant Science and Microbiology, Ohnhorststraße 18, D-22609 Hamburg, Germany

[2]Universität Jena, Institute of Ecology and Evolution, Philosophenweg 16, D-07743 Jena, Germany

[3]Bernhard Nocht Institute for Tropical Medicine, Bernhard-Nocht-Straße 74, D-20359 Hamburg, Germany

[4]Universidade Estadual Paulista "Júlio de Mesquita Filho", Instituto de Biociências, Departamento de Botânica, Av. 24 A 1515, Bela Vista, Rio Claro, São Paulo, CEP 13506-900, Caixa Postal 199, Brazil

Table S1. Structure of intron-containing genes in seven complete plastomes of *Ocotea* species.

Region	Gene	Exon 1, bp	Intron 1, bp	Exon 2, bp	Intron 2, bp	Exon 3, bp
LSC	*atpF*	145	726	410	-	-
	clpP	70	776	293	655	246
	rpl2	391	676	431	-	-
	rpl16	9	970	396	-	-
	rps16	43	847	227	-	-
	rpoC1	432	720	1620	-	-
	trnG-UCC	24	747	48	-	-
	trnK-UUU	37	2513	35	-	-
	trnL-UAA	35	479	50	-	-
	trnV-UAC	39	590	35	-	-
	pafI	126	735	228	732	153
IR	*ndhB*	758	702	756	-	-
	rps12	114	536	231	-	27
	trnA-UGC	38	798	35	-	-
	trnI-GAU	42	939	35	-	-
SSC	*ndhA*	553	1121	539	-	-

Table S2. AT and GC content in seven complete plastomes of *Ocotea* species.

Region	Nucleotide	*O. aciphylla* %	*O. daphnifolia* %	*O. foetens* %	*O. guianensis* %	*O. odorifera* %	*O. porosa* %	*O. tabacifolia* %
**LSC**	**A**	30.3	30.3	30.4	30.3	30.3	30.3	30.4
	**C**	19.4	19.4	19.3	19.4	19.4	19.4	19.4
	**G**	18.6	18.6	18.6	18.6	18.6	18.6	18.6
	**T**	31.7	31.7	31.7	31.7	31.7	31.7	31.6
	**GC**	38.0	38.0	37.9	38.0	38.0	38.0	38.0
**IRa**	**A**	28.3	28.3	28.3	28.3	28.3	28.3	28.3
	**C**	21.1	21.1	21.1	21.1	21.1	21.1	21.1
	**G**	23.4	23.4	23.4	23.4	23.4	23.4	23.4
	**T**	27.2	27.2	27.2	27.2	27.2	27.2	27.2
	**GC**	44.4	44.4	44.4	44.4	44.4	44.4	44.4
**IRb**	**A**	27.3	27.2	27.3	27.2	27.2	27.3	27.2
	**C**	23.4	23.4	23.4	23.4	23.4	23.4	23.4
	**G**	21.0	21.1	21.0	21.1	21.1	21.0	21.1
	**T**	28.3	28.3	28.3	28.3	28.3	28.3	28.3
	**GC**	44.4	44.4	44.4	44.4	44.4	44.4	44.4
**SSC**	**A**	32.9	32.9	32.9	32.9	32.9	32.9	32.9
	**C**	15.9	15.9	15.9	15.9	15.9	15.9	15.9
	**G**	18.1	18.1	18.1	18.1	18.1	18.1	18.1
	**T**	33.1	33.1	33.1	33.1	33.1	33.1	33.1
	**GC**	34.0	34.0	33.9	34.0	34.0	34.0	34.0
**CDS**	**A**	30.0	30.0	30.0	30.0	30.0	30.0	30.0
	**C**	19.7	19.7	19.7	19.7	19.7	19.7	19.7
	**G**	19.5	19.5	19.5	19.5	19.5	19.5	19.5
	**T**	30.8	30.8	30.8	30.8	30.8	30.8	30.8
	**GC**	39.2	39.2	39.2	39.2	39.2	39.2	39.2

Table S3. Relative synonymous codon usage (RSCU) in the plastomes of *Ocotea.*

		Ocotea aciphylla		***Ocotea daphnifolia***		***Ocotea foetens***		***Ocotea guianensis***		***Ocotea odorifera***		***Ocotea porosa***		***Ocotea tabacifolia***	
**AA**	**Codon**	Count	RSCU	Count	RSCU	Count	RSCU	Count	RSCU	Count	RSCU	Count	RSCU	Count	RSCU
**Phe**	**UUU**	818	1.09	818	1.09	812	1.09	818	1.09	818	1.09	818	1.09	819	1.09
	UUC	685	0.91	686	0.91	688	0.91	683	0.91	685	0.91	686	0.91	684	0.91
**Leu**	**UUA**	537	1.07	538	1.07	542	1.08	538	1.07	537	1.07	539	1.07	539	1.07
	**UUG**	704	1.4	704	1.4	696	1.39	702	1.39	702	1.4	702	1.4	703	1.4
	**CUU**	560	1.11	559	1.11	553	1.11	559	1.11	559	1.11	559	1.11	561	1.12
	CUC	362	0.72	359	0.71	360	0.72	359	0.71	359	0.71	360	0.72	359	0.71
	CUA	475	0.94	476	0.95	469	0.94	480	0.95	476	0.95	476	0.95	475	0.94
	CUG	380	0.76	382	0.76	380	0.76	382	0.76	382	0.76	381	0.76	380	0.76
**Ile**	**AUU**	819	1.19	821	1.19	814	1.19	822	1.19	821	1.19	818	1.19	818	1.19
	AUC	571	0.83	569	0.83	569	0.83	569	0.83	570	0.83	570	0.83	572	0.83
	AUA	678	0.98	677	0.98	670	0.98	677	0.98	678	0.98	677	0.98	678	0.98
**Met**	AUG	632	1	633	1	625	1	634	1	633	1	633	1	632	1
**Val**	**GUU**	438	1.26	436	1.26	437	1.27	435	1.26	437	1.26	438	1.27	436	1.26
	GUC	264	0.76	264	0.76	259	0.74	264	0.76	264	0.76	262	0.76	264	0.76
	**GUA**	392	1.13	393	1.14	388	1.13	392	1.13	392	1.13	393	1.14	389	1.13
	GUG	292	0.84	291	0.84	290	0.84	291	0.84	291	0.84	291	0.84	293	0.85
**Ser**	AGU	250	0.74	249	0.74	255	0.75	250	0.74	249	0.74	249	0.74	252	0.75
	AGC	150	0.44	149	0.44	153	0.45	150	0.44	148	0.44	148	0.44	149	0.44
	**UCU**	473	1.4	477	1.41	488	1.44	474	1.4	477	1.41	473	1.4	474	1.4
	**UCC**	376	1.11	374	1.11	372	1.09	375	1.11	374	1.11	372	1.1	373	1.1
	**UCA**	481	1.42	480	1.42	473	1.39	479	1.42	480	1.42	481	1.43	480	1.42
	UCG	299	0.88	298	0.88	298	0.88	300	0.89	300	0.89	299	0.89	299	0.89
**Pro**	**CCU**	265	1.04	266	1.04	271	1.06	267	1.05	266	1.04	266	1.04	267	1.04
	CCC	200	0.78	199	0.78	201	0.78	199	0.78	199	0.78	199	0.78	201	0.78
	**CCA**	309	1.21	310	1.22	307	1.2	306	1.2	310	1.22	311	1.22	308	1.2
	CCG	246	0.96	245	0.96	246	0.96	248	0.97	245	0.96	246	0.96	249	0.97
**Thr**	**ACU**	277	1.07	276	1.06	271	1.06	277	1.07	276	1.06	276	1.06	278	1.07
	ACC	222	0.86	223	0.86	221	0.86	219	0.85	224	0.86	223	0.86	220	0.85
	**ACA**	330	1.27	331	1.28	330	1.29	331	1.28	331	1.27	331	1.28	329	1.27
	ACG	208	0.8	208	0.8	205	0.8	209	0.81	208	0.8	208	0.8	208	0.8
**Ala**	**GCU**	251	1.33	252	1.34	243	1.31	251	1.33	252	1.34	251	1.33	253	1.34
	GCC	135	0.72	135	0.72	136	0.74	137	0.73	135	0.72	135	0.72	136	0.72
	**GCA**	232	1.23	232	1.23	227	1.23	232	1.23	232	1.23	232	1.23	234	1.23
	GCG	135	0.72	135	0.72	134	0.72	133	0.71	135	0.72	135	0.72	135	0.71
**Tyr**	**UAU**	695	1.31	694	1.31	699	1.3	695	1.31	694	1.31	695	1.31	695	1.31
	UAC	365	0.69	366	0.69	374	0.7	365	0.69	366	0.69	366	0.69	366	0.69
**His**	**CAU**	411	1.39	409	1.39	419	1.39	407	1.38	409	1.39	408	1.39	408	1.39
	CAC	180	0.61	180	0.61	182	0.61	181	0.62	180	0.61	180	0.61	179	0.61
**Gln**	**CAA**	548	1.19	547	1.18	544	1.19	545	1.18	547	1.18	547	1.18	545	1.18
	CAG	376	0.81	377	0.82	373	0.81	377	0.82	377	0.82	378	0.82	377	0.82
**Asn**	**AAU**	722	1.26	724	1.26	737	1.27	722	1.26	723	1.26	723	1.26	721	1.26

	AAC	424	0.74	426	0.74	423	0.73	425	0.74	426	0.74	424	0.74	424	0.74
**Lys**	**AAA**	920	1.23	919	1.23	919	1.24	927	1.24	923	1.24	923	1.24	923	1.24
	AAG	570	0.77	570	0.77	569	0.76	568	0.76	570	0.76	567	0.76	569	0.76
**Asp**	**GAU**	515	1.39	517	1.4	514	1.4	514	1.4	518	1.4	517	1.4	515	1.39
	GAC	225	0.61	224	0.6	221	0.6	220	0.6	224	0.6	223	0.6	224	0.61
**Glu**	**GAA**	671	1.26	668	1.25	670	1.26	671	1.26	668	1.25	671	1.26	667	1.25
	GAG	398	0.74	398	0.75	394	0.74	395	0.74	397	0.75	398	0.74	398	0.75
**Cys**	**UGU**	289	1.25	288	1.24	297	1.25	288	1.24	288	1.24	288	1.24	289	1.25
	UGC	175	0.75	175	0.76	179	0.75	175	0.76	175	0.76	175	0.76	175	0.75
**Trp**	**UGG**	497	1	496	1	496	1	498	1	496	1	497	1	495	1
**Arg**	**AGA**	463	1.86	459	1.83	473	1.86	461	1.85	459	1.83	461	1.84	462	1.85
	**AGG**	286	1.15	289	1.16	295	1.16	286	1.15	289	1.16	288	1.15	287	1.15
	CGU	161	0.65	163	0.65	166	0.65	163	0.65	163	0.65	163	0.65	163	0.65
	CGC	98	0.39	98	0.39	99	0.39	98	0.39	98	0.39	98	0.39	99	0.4
	**CGA**	281	1.13	286	1.14	280	1.1	283	1.13	286	1.14	285	1.14	285	1.14
	CGG	206	0.83	206	0.82	212	0.83	206	0.83	206	0.82	205	0.82	205	0.82
**Gly**	GGU	303	0.94	301	0.93	303	0.95	301	0.93	300	0.93	300	0.93	302	0.94
	GGC	174	0.54	177	0.55	175	0.55	176	0.55	177	0.55	178	0.55	175	0.54
	**GGA**	461	1.43	460	1.43	453	1.42	460	1.43	460	1.43	459	1.43	460	1.43
	**GGG**	351	1.09	352	1.09	349	1.09	354	1.1	352	1.09	351	1.09	354	1.1
*****	UAA	412	0.95	416	0.96	414	0.95	415	0.95	415	0.95	413	0.95	414	0.95
	**UAG**	495	1.14	496	1.14	492	0.86	494	1.13	495	1.14	495	1.14	494	1.14
	UGA	396	0.91	394	0.91	399	1.07	397	0.91	394	0.91	395	0.91	396	0.91

* = stop codon. The preferred codons are in bold (RSCU > 1.0)

Table S4. GC content and ENC of the protein-coding genes in seven *Ocotea* chloroplast genomes.

Taxa	ENC	CBI	SChi2	G+C2	G+C3	G+Cc	CC
Ocotea aciphylla	56.59	0.15	0.074	0.355	0.392	0.391	25,514
Ocotea daphnifolia	56.62	0.149	0.073	0.355	0.392	0.391	25,520
Ocotea foetens	56.62	0.151	0.074	0.356	0.392	0.391	25,503
Ocotea guianensis	56.61	0.15	0.073	0.355	0.392	0.391	25,509
Ocotea odorifera	56.61	0.15	0.073	0.355	0.392	0.391	25,520
Ocotea porosa	56.60	0.15	0.074	0.355	0.392	0.391	25,509
Ocotea tabacifolia	56.62	0.149	0.073	0.355	0.392	0.391	25,513

Abbreviations: CBI – Codon Bias Index; CC – codons count; G+C – content at coding positions: at second codon positions (G+C2); at (synonymous) third codon positions (G+C3s); at coding positions (G+Cc); ENC – effective number of codons; SChi2 – Scaled Chi-square.

Table S5. Counts of SSRs in LSC, SSC and IR regions of *Ocotea* plastomes.

	O. aciphylla			***O. daphnifolia***			***O. foetens***			***O. guianensis***			***O. odorifera***			***O. porosa***			***O. tabacifolia***		
	LSC	SSC	IR	LSC	SSC	IR	LSC	SSC	IR	LSC	SSC	IR	LSC	SSC	IR	LSC	SSC	IR	LSC	SSC	IR
A/T	46	10	1	45	9	1	45	14	1	47	13	1	47	9	1	46	11	1	48	13	1
C/G	3			1			2			4						2			3	1	
AG/CT	4			4			4			4			4			4			4		
AT/TA	4	2		4	1		5	1		4	1		4	1		4	1		4	1	
AAT/ATT	2	1		2	1		2	1		2	1		2	1		2	1		2	1	
AAAG/CTTT	1			1			1			1			1			1			1		
AAAT/ATTT	2		1	2		1	2		1	2		1	2		1	2		1	2		
AACT/AGTT	1			1			1			1			1			1			1		
AATG/ATTC	1			1			1			1			1			1			1		
AATT/AATT																			1		
ACAT/ATGT	1			1			1			1			1			1			1		
AACCT/AGGTT										1									1		
AAATC/ATTTG		1						1			1										
AAATAG/ATTTCT	1						1			1						1			1		
AATACT/AGTATT																		1			
Total	66	14	2	62	11	2	65	17	2	69	16	2	63	11	2	65	13	2	70	16	1

Table S6. Chloroplast genome sizes and numbers of genes in Lauraceae.

Group	Species	Reference	Genome size [bp]	LSC size [bp]	IR size [bp]	SSC size [bp]
	Caryodaphnopsis henryi Airy Shaw, ***C. malipoensis*** B. Liu, Y. Yang & K. P. Ma<br>***C. tonkinensis*** (Lecomte) Airy Shaw	Song *et al.*[25]<br>Song *et al.*[28]	149,239–154,938<br>148,829	86,035–91,901<br>91,762	20,036–25,601<br>19,695	17,266–17,701<br>17,677
	Cassytha filiformis L., ***C. capillaris*** Meisn.	Song *et al.*[25]	114,623–114,963	–	–	–
Cinnamomeae	***Cinnamomum micranthum*** (Hayata) Hayata, ***C. kanehirae*** Hayata<br>***C. camphora*** (L.) J. Presl<br>***C. aromaticum*** Nees, ***C. bodinieri*** H. Lév., ***C. burmanni*** (Nees & T. Nees) Blume, ***C. heyneanum*** Nees	Wu *et al.*[29]<br>Chen *et al.*[16]<br>Song *et al.*[28]	152,675–152,700<br>152,570<br>152,679–152,775	93,643–93,662<br>93,705<br>93,588–93,712	20,069–20,106<br>19,886<br>20,066–20,096	18,845–18,875<br>19,093<br>18,819–18,903
	Sassafras tzumu (Hemsl.) Hemsl.	Song et al.[25]	151,798	92,752	20,096	18,854
	Ocotea aciphylla (Nees & Mart.) Mez, ***O. daphnifolia*** (Meisn.) Mez, ***O. foetens (Aiton) Baill., O. guianensis Aubl., O. odorifera (Vell.) Rohwer, O. porosa*** (Nees & Mart.) Barroso, ***O. tabacifolia*** (Meisn.) Rohwer	this study	152,630–152,685	93,815–93,859	20,009–20,015	18,775–18,818
Cryptocaryeae	***Beilschmiedia pauciflora*** H. W. Li, ***B. tungfangensis*** S. K. Lee & L. F. Lau<br>***B. fasciata*** H. W. Li, ***B. robusta*** C. K. Allen, ***B. rufohirtella*** H. W. Li, ***B. turbinata*** Bing Liu & Y. Yang	Song *et al.*[25]<br>Song *et al.*[28]	157,901–158,530<br>158,337–158,417	88,351–88,673<br>89,273–89,275	25,473–25,496<br>25,445–25,488	18,233–18,236<br>18,173–18,216
	Cryptocarya chinensis (Hance) Hemsl., ***C. hainanensis*** Merr.<br>***C. chingii*** W. C. Cheng, ***C. densiflora*** Blume, ***C. wrayi*** Gamble, ***C. yunnanensis*** H. W. Li	Song *et al.*[25]<br>Song *et al.*[28]	157,145–157,675<br>157,132–157,739	89,002–89,199<br>88,984–89,263	24,621–24,627<br>24,621–24,624	18,901–19,222<br>18,897–19,234
	Endiandra globosa Maiden & Betche, ***E. discolor*** Benth.<br>***E. dolichocarpa*** S. K. Lee & Y. T. Wei, ***E. muelleri*** Meisn.	Rossetto *et al.*[53]<br>Song *et al.*[28]	158,567–158,585<br>158,571–158,610	–<br>89,317–89,321	–<br>25,507–25,522	–<br>18,236–18,249
	Eusideroxylon zwageri Teijsm. & Binn.	Song *et al.*[25]	157,577	89,231	24,717	18,912
	Syndiclis anlungensis H. W. Li, ***S. marlipoensis*** H. W. Li	Song *et al.*[28]	158,537–158,573	89,359–89,378	25,491–25,499	18,196–18,197
Laureae	***Actinodaphne trichocarpa*** C. K. Allen<br>***A. cupularis*** (Hemsl.) Gamble, ***A. hainanensis*** Merr., ***A. pilosa*** (Lour.) Merr., ***A. rugosa*** Merr. & Chun, ***A. yunnanensis*** Kosterm.	Song *et al.*[25]<br>Song *et al.*[28]	152,739<br>152,720–152,848	93,783<br>93,724–93,798	20,078<br>20,016–20,093	18,800<br>18,816–18,910
	Iteadaphne caudata (Nees) H. W. Li	Song *et al.*[28]	152,370	93,455	20,089	18,791
	Laurus nobilis L.	Zhang (unpubl.)<br>Song *et al.*[28]	152,750<br>152,608	–<br>93,516	–<br>20,061	–<br>18,970
	Lindera benzoin (L.) Blume, ***L. communis*** Hemsley, ***L. glauca*** (Siebold & Zuccarini) Blume, ***L. latifolia*** Hook. f., ***L. megaphylla*** Hemsley, ***L. metcalfiana* var. *dictyophylla*** (C. K. Allen) H. P. Tsui, ***L. nacusua*** (D. Don) Merr., ***L. obtusiloba*** Blume, ***L. robusta*** (C. K. Allen) H. P. Tsui	Zhao *et al.*[33]	152,211–152,968	93,573–93,888	20,048–20,061	18,336–18,948

	Litsea glutinosa (Lour.) C. B. Rob.<br>***L. cubeba*** *(Lour.)* Pers., ***L. glutinosa*** (Lour.) C. B. Rob., ***L. panamanja*** (Buch.–Ham. ex Nees) Hook. f**.**, ***L. pierrei*** Lecomte, ***L. tsinlingensis*** Y. C. Yang & P. H. Huang<br>***L. acutivena*** Hayata, ***L. dilleniifolia*** P.Y. Pai & P.H. Huang, ***L. elongata*** (Nees) J. D. Hooker, ***L. glutinosa*** (Lour.) C. B. Rob, ***L. mollis*** Hemsl., ***L. monopetala*** (Roxburgh) Persoon, ***L. pungens*** Hemsl., ***L. szemaois*** (H. Liu) J. Li & H.W. Li	Hinsinger *et al.*[17]<br>Song *et al.*[28]<br>Xiao *et al.*[30]	152,618<br>152,424–152,782<br>152,132–152,793	93,690<br>93,517–93,812<br>93,119–93,827	20,063<br>20,042–20,066<br>20,062–20,131	18,802<br>18,823–18,972<br>18,799–18,936
	Neolitsea sericea (Blume) Koidz.<br>***N. chui*** Merr., ***N. oblongifolia*** Merr. & Chun	Song *et al.*[25]<br>Song *et al.*[28]	152,442<br>152,727–152,747	93,803<br>93,836–93,843	20,067<br>20,016	18,505<br>18,808–18,821
	Parasassafras confertiflorum (Meisn.) D. G. Long	Liao *et al.*[19]	152,555	93,604	20,079	18,793
	Neocinnamomum caudatum (Nees) Merr., ***N. lecomtei*** H. Liu	Song *et al.*[25]	150,838–150,842	91,881–91,912	20,257	18,412–18,447
**Perseeae**	***Alseodaphne gracilis*** Kosterm., ***A. huanglianshanensis*** H. W. Li & Y. M. Shui, ***A. semecarpifolia*** Nees	Song *et al.*[26]	153,051–153,099	93,821–93,847	20,280–20,285	18,670–18,682
	Dehaasia incrassata (Jack) Kosterm.	Song *et al.*[28]	152,723	93,712	20,056	18,899
	Machilus balansae (Airy Shaw) F. N. Wei & S. C. Tang, ***M. yunnanensis*** Lecomte<br>***M. fasciculata*** H. W. Li, ***M. pauhoi*** Kaneh., ***M. thunbergii*** Siebold & Zucc.	Song *et al.*[23]<br>Song *et al.*[28]	152,622–152,721<br>152,550–152,620	93,675–93,676<br>93,650–93,670	20,074<br>20,050–20,074	18,799–18,897<br>18,800–18,805
	Nothaphoebe umbelliflora (Blume) Blume	Song *et al.*[28]	152,832	93,747	20,078	18,870
	Persea americana Mill.<br>***P. americana*** var. ***drymifolia*** (Schltdl. & Cham.) S. F. Blake<br>***P. borbonia*** (L.) Spreng.	Song *et al.*[24]<br>Song *et al.*[28]	152,723<br>152,862<br>152,394	93,795<br>93,845<br>93,314	20,052<br>20,196<br>20,076	18,824<br>18,599<br>18,928
	Phoebe omeiensis R. H. Miao, ***Ph. sheareri*** (Hemsl.) Gamble<br>***Ph. lanceolata*** (Wall. ex Nees) Nees	Song *et al.*[27]<br>Song *et al.*[28]	152,855–152,876<br>152,809	93,775<br>93,767	20,076–20,093<br>20,073	18,915–18,928<br>18,896

Table S7. Taxa, origin and vouchers of *Ocotea* species in this study.

Species	Provenance	Date	Voucher	Acc. No.	det. by	GenBank Acc. No.
O. aciphylla (Nees & Mart.) Mez	Brazil: Espírito Santo	09 Sep 2011	*Moraes 3205*	HRCB 56100	P. Moraes	OM135246
O. daphnifolia (Meisn.) Mez	Brazil: Espírito Santo	11 Sep 2011	*Moraes 3239*	HRCB 56134	J. Rohwer	OM135247
O. foetens (Aiton) Baill.	Germany: Botanical Garden Berlin (origin unknown)	27 Mar 2019*	*B Gard. Herb. 40713*	300419680, IPEN XX-0-B-3004196	B. Leuenberger	OM135248
O. guianensis Aubl.	Brazil: Mato Grosso	16 Nov 1996	*Hatschbach 65624*	HBG w/o no.	G. Hatschbach	OM135249
O. odorifera (Vell.) Rohwer	Brazil: Espírito Santo	11 Sep 2011	*Moraes 3247*	HRCB 56142	P. Moraes	OM135250
O. porosa (Nees & Mart.) Barroso	Brazil: São Paulo	29 Sep 2011	*Moraes 3375*	HRCB 56179	P. Moraes	OM135251
O. tabacifolia (Meisn.) Rohwer	Brazil: São Paulo	01 Sep 2011	*Moraes 3357*	HRCB 56167	P. Moraes	OM135252

*date of collection of leaf material by JGR

Table S8. GenBank accession numbers and references for Lauraceae chloroplast genome sequences downloaded for this study.

**Taxon**	**GenBank accession number**	**Reference**
Actinodaphne lancifolia (Blume) Meisn.	NC045251	Jo *et al.*[18]
Actinodaphne obovata (Nees) Blume	NC050360	Xiao *et al.*[30]
Actinodaphne trichocarpa C.K. Allen	MF939342	Song *et al.*[25]
Alseodaphne gracilis Kosterm.	MG407593	Song *et al.*[26]
Alseodaphne huanglianshanensis H. W. Li & Y. M. Shui	MG407595	Song *et al.*[26]
Alseodaphne semecarpifolia Nees	MG407594	Song *et al.*[26]
Cinnamomum aromaticum Nees	NC046019	Gui (unpubl.)
Cinnamomum camphora (L.) J. Presl	LC228240	Chen *et al.*[16]
Cinnamomum kanehirae Hayata	KR014245	Wu *et al.*[29]
Cinnamomum kotoense Kanehira & Sasaki	NC050346	Yuan *et al.*[31]
Cinnamomum micranthum (Hayata) Hayata	KT833081	Wu *et al.*[29]
Cinnamomum parthenoxylon (Jack) Meisn.	MH050971	Wu *et al.*[29]
Cinnamomum pittosporoides Handel-Mazzetti	NC048978	Zhou *et al.*[54]
Cinnamomum verum J. Presl	KY635878	Rabah *et al.*[39]
Cinnamomum yabunikkei H. Ohba	NC044864	Ren (unpubl.)
Laurus nobilis L.	NC034700	Zhang (unpubl.)
Lindera aggregata (Sims) Kosterm.	NC045252	Jo *et al.*[18]
Lindera angustifolia W.C.Cheng	NC045253	Jo *et al.*[18]
Lindera benzoin (L.) Blume	MH220730	Zhao *et al.*[33]
Lindera chunii Merr.	NC045254	Jo *et al.*[18]
Lindera communis Hemsl.	MH220731	Zhao *et al.*[33]
Lindera erythrocarpa Makino	NC045256	Jo *et al.*[18]
Lindera floribunda (C. K. Allen) H. P. Tsui	NC045257	Jo *et al.*[18]
Lindera glauca (Siebold & Zucc.) Blume	MF188124	Xiong *et al.*[55]
Lindera latifolia Hook. f.	MH220733	Zhao *et al.*[33]
Lindera megaphylla Hemsl.	MH220734	Zhao *et al.*[33]
Lindera metcalfiana var. *dictyophylla* (C. K. Allen) H. P. Tsui	MH220735	Zhao *et al.*[33]
Lindera nacusua (D. Don) Merr.	MH220736	Zhao *et al.*[33]
Lindera neesiana (Wallich ex Nees) Kurz	NC045261	Jo *et al.*[18]
Lindera obtusiloba Blume	MH220737	Zhao *et al.*[33]
Lindera praecox (Siebold & Zuccarini) Blume	NC045263	Jo *et al.*[18]
Lindera pulcherrima var. *attenuate* C.K. Allen	MG581450	Jo *et al.*[18]
Lindera reflexa Hemsl.	NC045264	Jo *et al.*[18]
Lindera robusta (C. K. Allen) H. P. Tsui	MH220738	Zhao et al.[33]
Lindera rubronervia Gamble	NC045265	Jo *et al.*[18]
Lindera sericea (Siebold & Zucc.) Blume	NC045266	Jo *et al.*[18]
Litsea acutivena Hayata	NC050362	Xiao *et al.*[30]
Litsea cubeba (Lour.) Pers.	NC048954	Wang *et al.*[55]
Litsea dilleniifolia P.Y. Pai & P.H. Huang	NC050363	Xiao *et al.*[30]

Litsea elongata (Nees) J. D. Hooker	NC050364	Xiao *et al.*[30]
Litsea garrettii Gamble	MN698967	Qiu *et al.*[22]
Litsea glutinosa (Lour.) C. B. Rob.	KU382356	Hinsinger *et al.*[17]
Litsea japonica (Thunb.) Juss.	NC045267	Jo *et al.*[18]
Litsea mollis Hemsl.	NC050366	Xiao *et al.*[30]
Litsea monopetala (Roxburgh) Persoon	NC050367	Xiao *et al.*[30]
Litsea pungens Hemsl.	NC050368	Xiao *et al.*[30]
Litsea szemaois (H. Liu) J. Li & H.W. Li	NC050369	Xiao *et al.*[30]
Machilus balansae (Airy Shaw) F. N. Wei & S. C. Tang	KT348517	Song *et al.*[23]
Machilus pauhoi Kaneh.	NC038203	Zhao *et al.*[33]
Machilus thunbergii Siebold & Zucc.	NC038204	Zhao *et al.*[33]
Machilus yunnanensis Lecomte	KT348516	Song *et al.*[23]
Nectandra angustifolia (Schrad.) Nees & Mart.	MF939340	Song *et al.*[25]
Neolitsea pallens (D. Don) Momiyama & H. Hara	NC050370	Xiao *et al.*[30]
Parasassafras confertiflorum (Meisn.) D.G. Long	NC042696	Liao *et al.*[19]
Persea americana Mill.	KX437771	Song *et al.*[24]
Phoebe bournei (Hemsl.) Y. C. Yang	MF315088	Li (unpubl.)
Phoebe chekiangensis P. T. Li	KY346511	Li *et al.*[40]
Phoebe neurantha (Hemsl.) Gamble	MH394353	Zeng *et al.*[56]
Phoebe omeiensis R. H. Miao	KX437772	Song *et al.*[27]
Phoebe puwenensis W.C. Cheng	NC050171	Zhang *et al.*[32]
Phoebe sheareri (Hemsl.) Gamble	KX437773	Song *et al.*[27]
Phoebe zhennan S. K. Lee & F. N. Wei	MF315089	Liu *et al.*[40]
Sassafras tzumu (Hemsl.) Hemsl.	MF939339	Song *et al.*[25]

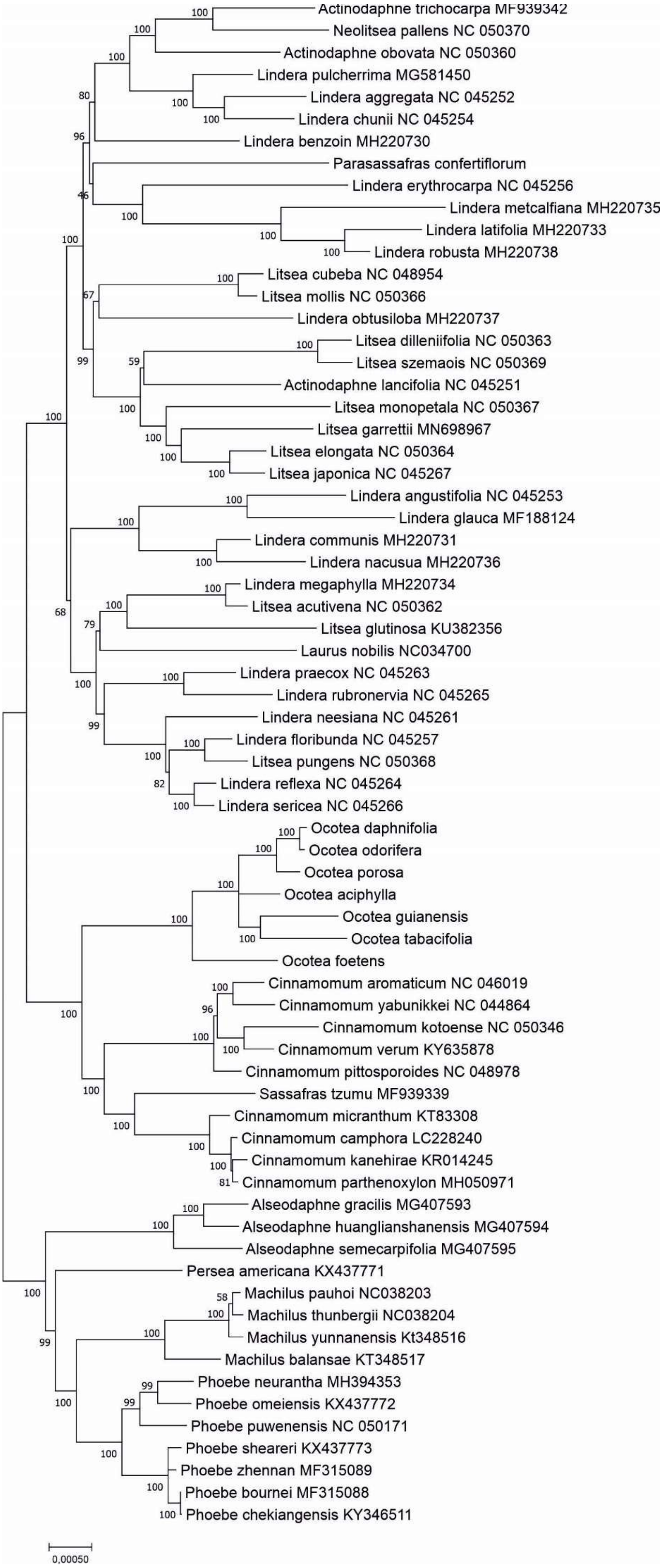
Actinodaphne trichocarpa MF939342
Neolitsea pallens NC 050370
Actinodaphne obovata NC 050360
Lindera pulcherrima MG581450
Lindera aggregata NC 045252
Lindera chunii NC 045254
Lindera benzoin MH220730
Parasassafras confertiflorum
Lindera erythrocarpa NC 045256
Lindera metcalfiana MH220735
Lindera latifolia MH220733
Lindera robusta MH220738
Litsea cubeba NC 048954
Litsea mollis NC 050366
Lindera obtusiloba MH220737
Litsea dilleniifolia NC 050363
Litsea szemaois NC 050369
Actinodaphne lancifolia NC 045251
Litsea monopetala NC 050367
Litsea garrettii MN698967
Litsea elongata NC 050364
Litsea japonica NC 045267
Lindera angustifolia NC 045253
Lindera glauca MF188124
Lindera communis MH220731
Lindera nacusua MH220736
Lindera megaphylla MH220734
Litsea acutivena NC 050362
Litsea glutinosa KU382356
Laurus nobilis NC034700
Lindera praecox NC 045263
Lindera rubronervia NC 045265
Lindera neesiana NC 045261
Lindera floribunda NC 045257
Litsea pungens NC 050368
Lindera reflexa NC 045264
Lindera sericea NC 045266
Ocotea daphnifolia
Ocotea odorifera
Ocotea porosa
Ocotea aciphylla
Ocotea guianensis
Ocotea tabacifolia
Ocotea foetens
Cinnamomum aromaticum NC 046019
Cinnamomum yabunikkei NC 044864
Cinnamomum kotoense NC 050346
Cinnamomum verum KY635878
Cinnamomum pittosporoides NC 048978
Sassafras tzumu MF939339
Cinnamomum micranthum KT83308
Cinnamomum camphora LC228240
Cinnamomum kanehirae KR014245
Cinnamomum parthenoxylon MH050971
Alseodaphne gracilis MG407593
Alseodaphne huanglianshanensis MG407594
Alseodaphne semecarpifolia MG407595
Persea americana KX437771
Machilus pauhoi NC038203
Machilus thunbergii NC038204
Machilus yunnanensis Kt348516
Machilus balansae KT348517
Phoebe neurantha MH394353
Phoebe omeiensis KX437772
Phoebe puwenensis NC 050171
Phoebe sheareri KX437773
Phoebe zhennan MF315089
Phoebe bournei MF315088
Phoebe chekiangensis KY346511
0,00050

Figure S1. Phylogenetic tree inferred by using the Maximum Likelihood method based on the Tamura-Nei model. The tree with the highest log likelihood (-334239.90) is shown. The percentage of trees in which the associated taxa clustered together is shown next to the branches.

Additional references in the Supplementary Data

(References 1–52 cited in the article)

53. Rossetto, M., Kooyman, R., Yap, J. Y. & Laffan, S. W. From ratites to rats: the size of fleshy fruits shapes species' distributions and continental rainforest assembly. *Proc. R. Soc. B* **282**, 20151998, 10.1098/rspb.2015.1998 (2015).

54. Zhou, X. L. *et al.* The complete chloroplast genome of *Cinnamomum pittosporoides* reveals its phylogenetic relationship in Lauraceae. *Mitochondrial DNA B Resour.* **4**(2), 3246–3247, 10.1080/23802359.2019.1669503 (2019).

55. Xiong, B., Zhang, L., Xie, L., Dong, Sh. & Zhang, Z. Complete chloroplast genome of a valuable economic tree, *Lindera glauca* (Lauraceae) and comparison with its congeners. *Pak. J. Bot.* **50**(6), 2189–2196, http://www.pakbs.org/pjbot/papers/1531142633.pdf (2018).

56. Zeng, C. X. *et al.* Genome skimming herbarium specimens for DNA barcoding and phylogenomics. *Plant Methods* **14**, 43, https://doi.org/10.1186/s13007-018-0300-0 (2018).
